# Supplementary material for: EXTending availability of self-management structured EducatioN programmes for people with type 2 Diabetes in low-to-middle income countries (EXTEND)—a feasibility study in Mozambique and Malawi
Source: BMJ Open. 2021 Sep 21;11(9):e047425. doi: 10.1136/bmjopen-2020-047425 (PMC8458338; doi:10.1136/bmjopen-2020-047425)
Supplement: Supplementary data [file bmjopen-2020-047425supp001.pdf]

EXTEND Supplementary material 1: CONSORT diagram

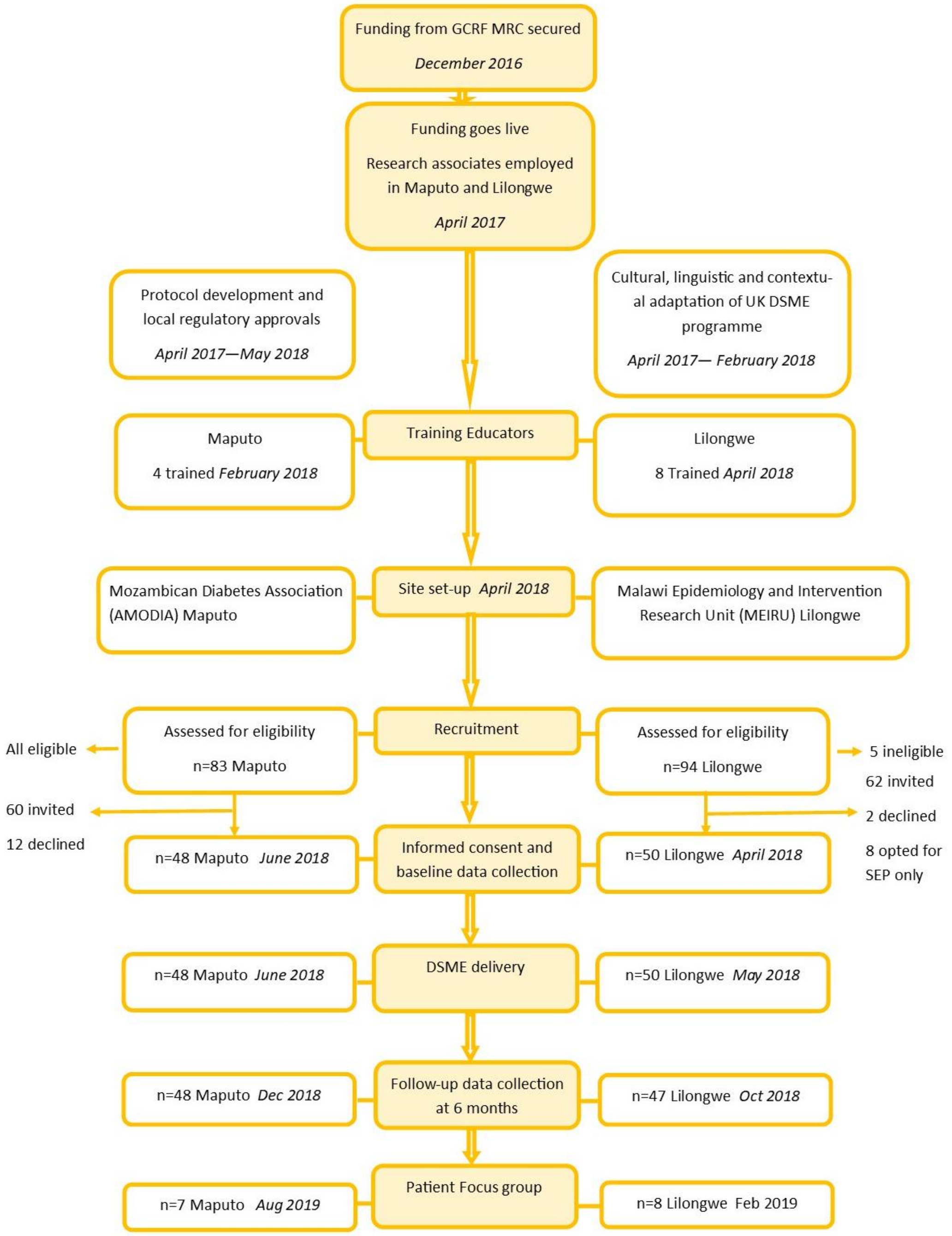

Brady EM, *et al. BMJ Open* 2022; 11:e047425. doi: 10.1136/bmjopen-2020-047425

EXTEND Supplementary material 2: DESMOND programme and EXTEND adaptations

The DESMOND curriculum is broken down into various sessions to help build on the persons understanding of the condition.

The sessions are as follows:
Section A Introduction and Housekeeping

Section B The participant story

Section C Type 2 diabetes and Glucose

Section D Monitoring type 2 diabetes

Section E Food and glucose control

Section F Reflections

Section G Reflections if delivering over 2 days

Section H Long term effects of type 2 diabetes

Section I Physical activity

Section j Food and health

Section K Self-management plan

Section L questions and future care

**Item**	**Adaptation**
Language	Maputo - Portuguese<br>Lilongwe – Chicheŵa<br>More images used as opposed to words driven by expected low literacy levels (outside the urban areas)<br>The handouts were able to be seen via a mobile device as printing options were limited to have paper copy of resources
Terminology	Glucose referred to as sugar throughout as this was a more familiar term
Images	Removal and replacement of image irrelevant to local people. This included an image of hovering, and replace with brush for sweeping floors. Add image of washing line and washing clothes by hand because there are no machines available/used.<br><br>Multiple images were provided for blood pressure monitoring and overweight with the group selecting the most appropriate ones that happened to differ from DESMOND.
Diabetes complications<br>Depression	The concept of 'depression' does not exist in these two cultures 'low feelings' / 'low mood' replaced depression. New innovative resources were developed for helping to score the low moods so these were then talked about during the session
Diabetes complications<br>Hypercholesterolemia	LDL should be used & not HDL as the evidence for CV risk is based on LDL levels not HDL.
Diabetes Complications<br>Erectile dysfunction<br>Retinopathy	If a male has erectile dysfunction his partner/wife will believe that this is because he is being unfaithful. This leads to wider martial problems. Therefore a session was added to explain what this health issues is, why it happens and what can be done to improve it.<br><br>Various tools were used when delivering the complications session to include a sieve to explain more fully about retinopathy. The access to routine retinal screening was an issue so again it was about supporting the person living with diabetes to be able to change those things that they had control over or indeed had access to via their local health care system.
Traditional medicine/religious beliefs	A session was added to allow discussion of this topic to explore traditional and folklore and how to understand what would work to manage diabetes and what was not considered relevant to support diabetes. For example in some villages hypoglycaemic events are viewed as witchcraft – in which the person has been possessed and therefore it is a taboo subject.<br><br>Some individuals may seek out the help of a "local Dr /elder in the community "in search of a cure but this isn't as a long term thing as they are expected to pay for this advice and may be given herbs or spices.
Pictorial representation	Signs/symptoms of type 2 diabetes, causes, ways to manage blood sugar, symptoms of highs and lows, how to treat lows(hypos), ways to manage weight, reduce cholesterol, blood pressure,

<table>
<tr><td></td><td colspan="2">smoking cessation, improve mood, benefits of activity, looking after feet, good and bad fats, a pictorial health profile and action plan</td></tr>
<tr><td>Food sessions</td><td colspan="2">Laminated pictures of food selected and sources by patients and educators examples provided below.</td></tr>
<tr><td></td><td>Malawi food examples
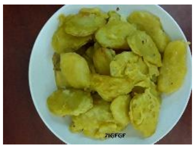
Zigege
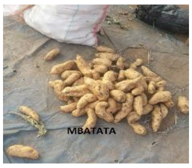

Mbatata
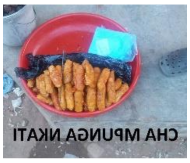
Cha mpunga nkati
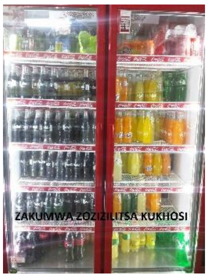

Isohkuk astilizizoz Awmukaz</td><td>Mozambique food examples
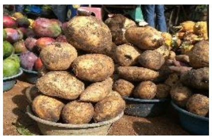
Batata
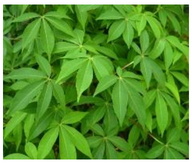
Cassava leaves
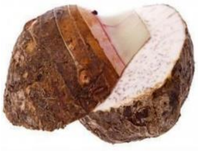
Inhame
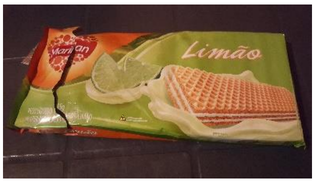

Wafer</td></tr>
<tr><td>Session E Food and glucose control</td><td colspan="2">100 Kcal game chickpeas were used instead of a kit-kat. The discussions were the same except the groups were how long it would take them to walk off each of the items

Carbohydrate and weight management there were no other changes as the basic carbohydrate foods were eaten in both countries so the activity and discussions didn't differ</td></tr>
<tr><td>Session J Food and health</td><td colspan="2">The message regarding fats was simplified to good and bad fat and included pictures of the fats used locally which were laminated. These were included in the fat continuum.</td></tr>
<tr><td>Delivery structure</td><td colspan="2">2*3 hour session was selected by user groups. No whole day sessions were provided.</td></tr>
</table>

Brady EM, *et al. BMJ Open* 2022; 11:e047425. doi: 10.1136/bmjopen-2020-047425

EXTEND Supplementary material 3: Training report Malawi

Running order of the training programme

<u>Core day 1</u>: With Interpreter and Project Coordinator
9.00 - 12.00 Covered: Format of training, Philosophy,Theories, Facilitation Skills and Educator behaviour, Observation tools
12.00 – Onwards: Trainers to prepare room and all resources for Training

<u>Core day 2</u>: With Educators, Interpreter and Project Coordinator
09.00 Introductions/Housekeeping/Outline of week.
09.20 Educators introduce themselves/current role and previous experience of delivering education
09.40 Break
10.00 Your role in this study - delivered by Project Supervisor
10.40 Exploring Philosophy underpinning SEP
11.00 Linking underpinning theories to sessions in the SEP
12.30 Lunch
13.15 Focus on Educator facilitation skills
14.00 Focus on Educator behavior
14.40 Introduction to the curriculum
15.40 Close – Trainers prepare room for tomorrow

<u>Core day 3</u> with Educators, Interpreter and Project Coordinator
8.30 Welcome back/Housekeeping – what do they remember from yesterday?
09.00 Walk through the Programme – Sessions A-F
10.30 Break
10.40 Walk through the Programme continued– Sessions G- L
12.15 Lunch
13.15 Preparation and Practice – Educators plan their delivery
And introduction of observation tools
15.30 Close and Evaluations

<u>Core Day 4</u>: With Educators, Interpreter and Project Coordinator
08.30 Welcome back/Housekeeping/Purpose of today
08.40 Preparation and practice for delivery
12.00 Lunch
13.00 Preparation of room and further practice
15.30 Close

<u>Delivery to patients – Day 1</u> with Educators, Interpreter and Project Coordinator
08.00 Educators and Trainers arrive to prepare for session
08.30 Patients arrive
09.00 Programme delivery and observation begins
12.30 End of part 1 of programme - Lunch
13.30 Educator feedback and preparation and practice for next day
15.30 Close

<u>Delivery to patients – Day 2</u> with Educators, Interpreter and Project Coordinator
08.00 Educators and Trainers arrive to prepare for session
08.30 Patients arrive
09.00 Programme delivery and observation begins
12.30 End of part 2 of programme
13.30 Educator feedback and ongoing support of Educator development
15.30 Close

Brady EM, *et al. BMJ Open* 2022; 11:e047425. doi: 10.1136/bmjopen-2020-047425

Introduction

As part of the EXTEND Study 2 Trainers from the Leicester Diabetes Centre in the United Kingdom (UK) visited Lilongwe in June 2017 to deliver a UK based SEP to patients and to take feedback on how it could be adapted and translated to be more culturally sensitive to the target population in Lilongwe.

In February 2018 the 2 Trainers returned with the adapted curriculum and its associated resources with the intention of training local Educators to be able to deliver to 50 patients during the feasibility study.

It was also an opportunity to identify if the adaptations had been correctly interpreted following the last visit and to explore with Hazel Namadingo about patient engagement to encourage attendance to the programme and ideas on sustainability should the programme go beyond the pilot.

Training Plan

The training had 5 components:

1. Training for the Interpreter and Project Coordinator in the background to the programme and quality assurance.

As the training had to be delivered via an Interpreter it was important that separate training for the Interpreter and the project lead in the underpinning philosophy of the programme was built into the Training Plan. The interpreter and the project lead were also trained on how to use the observation tools as part of the Quality Assurance model for this study.

2. Training for the Educators in the background to the programme

Training the Educators so that they had a clear understanding of how the structured written curriculum and resources should be used. This involved exploring the importance of the underpinning philosophy of the programme and giving them a realistic understanding on the adult learning theories upon which the programme is based and why the sessions have been put together the way they have been.

To complement this - time was also included on facilitation skills and Educator behaviors which would support delivery of the programme using the underpinning philosophy.

3. Training the Educators in how to use the SEP curriculum and associated resources.

All but 2 of the Educators that were to be trained had not observed delivery to patients during the last visit so it was important for the Trainers to ‘walk the Educators through’ each session of the programme, modeling each session to allow for discussion on potential challenges that may occur during delivery and exploring potential strategies to deal with challenges.

4. Supporting Educators to prepare and practice delivery to patients.

Of the 8 Educators who were trained over 3 days, 4 were selected to go forward to deliver in the pilot.

Of these 4 Educators, 2 were patients with diabetes and 2 were Nurses.

These 4 Educators went on to have another days training to allow them to prepare and practice for delivery to patients

Brady EM, *et al. BMJ Open* 2022; 11:e047425. doi: 10.1136/bmjopen-2020-047425

5. Supporting Educators actually delivering to patients via observation and feedback

Although the programme is intended to be delivered by 2 Educators, working together to support each other - for the purposes of the training and because the Educator team had a mix of Health Care Professionals and patients it was decided that they would deliver as a team of 4, at least for the purposes of the training as their practice delivery came the day after completion of training.

The Educators should be able to deliver as a pair for the feasibility study but it’s recommended that they should be paired as Health Care Professional with a patient. Should they wish to deliver as a team of 4 this should not affect the study but would obviously impact on Educator capacity.

The delivery of the programme to 10 patients took place over 2 half days and both the Trainers and the Project Coordinator used observational tools to help provide objective feedback to each Educator with regards to key content covered, time management, Educator talk time, the use of Educator specific behaviors and non-verbal behaviors during delivery that linked to the philosophy underpinning the programme

The Educators were also trained and encouraged to use self-reflection tools to support their onward development and were offered access to the Trainers for further support following the visit should that be required, via email or skype.

At the end of the pilot it is hoped themes from the Educators self-reflections can be summarised to identify any learning that could be incorporated into any future Educator training should the SEP be rolled out further.

The Trainers would encourage the Project Coordinator where possible to continue to use the observation sheets which measure key content covered, Educator specific behavior and time management whenever it’s feasible for her to observe future Educator delivery – If Possible observing 2 of the 5 SEPs as part of the feasibility study.

Evaluation of the Training - summary feedback received from the Educators

‘The training has been good – I liked your facilitation’

‘All sessions were great – we learnt new things that will help us teach others and you have taught us very well’

‘Our friends with type 2 diabetes will benefit a lot from this SEP and we will work hard to teach others so that type 2 diabetes can be managed

‘Thank you for this SEP – we have learned a lot and we will teach others’

‘The sessions have been good. Time was managed well – facilitators were very good and friendly’

Evaluation of the Educators first delivery to patients following training using observation tools – summary of results for all 4 Educators

1. Key Content covered – all 4 Educators covered all key content of the sessions they delivered.

Brady EM, *et al. BMJ Open* 2022; 11:e047425. doi: 10.1136/bmjopen-2020-047425

2. Specific Educator behaviors (e.g. Open questions/Reflections and Summaries) – all 4 Educators frequently used open questions, reflections and summaries as well as analogies , examples and visual aids to engage with patients to support systematic learning.

3. Nonverbal Educator behaviors (e.g. eye contact/smiling/open body language) –all 4 Educators displayed positive nonverbal behaviors to support patient engagement throughout all sessions.

4. Educator Talk Time using DESMOND Observation Tool – all 4 Educators achieved the talk time targets based on delivery in English even though they were delivering in Chichewa

5. Time management – the timing of session delivery was generally very well adhered to throughout the whole 6 hour programme.

Summary

The UK Training team adapted the training they normally deliver in the UK to concentrate on specific behaviors and facilitation skills they wanted the Educators to use to support the underpinning philosophy of the SEP when they delivered.They also built in more time for the Trainers to model each of the sessions to allow the curriculum to 'come to life'.

Although only 4 of the 8 Educators trained will be delivering in the pilot – the 4 who will not be will take their knowledge and awareness of the programme back into their local communities and local groups that they engage with . Hopefully these ambassadors will encourage patient recruitment to attend this SEP during the pilot and, if successful, potentially beyond.

By including the Project Coordinator in the training and in particular in the observations of delivery it was felt this would provide extra support in the Educators onward journey when the UK team had left.

In addition the Trainers wanted the Educators to take ownership of their curriculum and so Educators were encouraged to highlight if there were errors in the curriculum or where there were more suitable analogies, food examples etc. that would be more appropriate for their local audience.

What the team in Lilongwe did in planning for 2 practice deliveries to patients by the Educators – one whilst the UK Trainers were there and one a week later - will undoubtedly have supported Educators confidence for when they deliver in the pilot. In addition there were also plans for other ways the Educators could practice delivery in other areas in the time between completion of training and delivery in the pilot.

The Trainers will share these ideas with the other EXTEND site in Maputo, Mozambique.

Acknowledgements

We would like to thank Mia Crampin (Acting Director MEIRU) and Hazel Namadingo (Project Supervisor) for arranging the visit, including logistics and supporting our training plan throughout and beyond.

We would also like to thank Catherine Bamuya (Project Coordinator) for her enthusiasm particularly in the support she helped us with in the observations and to all the individuals who contributed to helping with interpreting throughout our visit especially Veronica.

Last but not least thanks to all the 8 Educators we trained for their enthusiasm and commitment who are moving forward to delivering in the pilot and to the patients who attended the SEP during our visit.

Appendices

Appendix 1

Part 2: Content Assessment Tool

Educator Name: ____________ Date: ____________

SESSION B: The Participant Story (40 mins)			
Start:	Finish:	Time taken:	✔ or ✖
Identifies individual participant stories by asking:			
How long do they believe they have had diabetes?			
How did they find out they had diabetes/any symptoms?			
What do they believe caused their diabetes?			
What do they believe are the long-term effects of having diabetes?			
What do they believe are the treatments for their diabetes?			
What is one key question, you would like answering before the end of the course?			
		Score	/6
Assessment Comments			

Appendix 2

Part 3: DESMOND Observational Tool (DOT)

Assessing Educator Talk Time Tool - **Must complete Session C or H plus one other from Sessions C, E, H, J or K**

Educator Name: Date:

DOT assessment			
Session:	Educator Talking:	Participant talking:	Miscellaneous:
Totals:	Total A:	Total B:	Total C:
(Total A) ÷ (Total A+B+C) = x 100 = Score: %			
Session:	Educator Talking:	Participant talking:	Miscellaneous:
Totals:	Total A:	Total B:	Total C:
(Total A) ÷ (Total A+B+C) = x 100 = Score: %			
Session:	Educator Talking:	Participant talking:	Miscellaneous:
Totals:	Total A:	Total B:	Total C:
(Total A) ÷ (Total A+B+C) = x 100 = Score: %			

Session Target	Educator Speaking Below:	Session Target	Educator Speaking Below:
C: Type 2 Diabetes and Glucose	65%	J: Focus on Fat	55%
E: Food Choices: Glycaemia and Insulin Resistance	55%	K: Diabetes Self-Management Plan	50%
H: Long-Term Effects of Type 2 Diabetes	65%		

Brady EM, et al. BMJ Open 2022; 11:e047425. doi: 10.1136/bmjopen-2020-047425

Appendix 3

Educator Self Reflection sheet

**What went well?**
**What didn't go so well?**
**What will you do about it?**

Appendix4

Training Evaluation Sheet

What session did you find most useful & why?
What would you have liked us to spend more time on?
Any other comments

Brady EM, *et al. BMJ Open* 2022; 11:e047425. doi: 10.1136/bmjopen-2020-047425

Supplementary material 4: Data collection procedures for demographic, clinical, bio-anthropometric characteristics and psychological wellbeing.

Medical History

Participants were advised to bring their health passports to both measurement sessions to assist with medical history. The following data were taken from the health passport and/or self-reported by the participant

Medical History	
Co-morbidities	Non-communicable Diseases Questionnaire (NCDQ) [1]
Diabetes Duration	Year of TD2 diagnosis for duration to be calculated (Q6042- Q6045 WHO Survey)[2]
Current medication	Taken from participant's health passports or self -report. Anti-viral medication use was to be collected on a separate CRF and stored separately to both the contacts form and main CRF (link anonymised using the unique Participant Identification number (PID)
Smoking status	Do you currently smoke any tobacco products such as cigarettes, cigars, or pipes?" and answer "Daily, yes not daily, not at all" (Q4000 WHO Survey)[2]
Alcohol	Alcohol Consumption captured using the eight questions in the STEPs survey [3]
Family history of diabetes	Provided list of family members (i.e. mother, father, siblings, paternal and maternal grandmother and grandfather) and asked whether any of them had diagnoses of T1D or T2D over their lifetime

Patient outcomes

Demographics	
Age	Years
Sex	Male or female
Ethnicity	*What is your [ethnic group / racial group / cultural subgroup / others] background*? Answers will come from a list of *relevant response options. (*Q1011 WHO Survey [2])
	Collect first language *"What is your mother tongue?" (*Q1000 of WHO Survey [2])
Socio-economic status	"What is the highest level of education you have completed?" (list of categories from Q1009 WHO Survey [2])
	Occupation (Q1012- Q1014 WHO Survey [2])
	Urban/rural home location will be ascertained based on address of participant

Bio- Anthropometric measurements	
Height	Measured to the nearest 0.1 cm using a portable stadiometer
Weight	Measured to the nearest 0.1 kg using a clinically approved weighing scale
Body mass index (BMI)	Calculated as weight (kg)/height (m^2)
Waist circumference	Measured with an inelastic anthropometry tape to the nearest 0.1 cm at the midpoint between the lower costal margin and iliac crest
Hip circumference	Measured at the level of the greatest protrusion of the gluteal (buttock) muscles whilst ensuring that the tape was not too tight or too loose, was lying flat on the skin, and horizontal. The participant stood erect with their weight evenly distributed on both feet and legs slightly parted, making sure not tense the gluteal (buttock) muscles

fasting venous blood sample	Taken by a trained Health Care Professional (HCP). Full-blood count, HbA1c, triglycerides and LDL cholesterol was measured from this sample
Blood pressure	Measured using an automated sphygmomanometer with an appropriate sized cuff while the patient was seated, and having rested quietly for 5 minutes. Three measurements were obtained for blood pressure with the average of the last two used in analysis

Health and wellbeing	
Depression	The Patient Health Questionnaire (PHQ-9) [4,5]
Diabetes related distress	PAID (Problem Areas in Diabetes Questionnaire (PAID) short form [6]
Quality of Life	The MOS short-form quality of life survey [7]
Mood	WHO-5 questionnaire commonly used to measure mental wellbeing [8]
Self-efficacy	The Self-efficacy for diabetes questionnaire is a reliable and valid 8-item scale tested in adult with diabetes [9]

Lifestyle behaviours	
Physical activity	Participants answered basic questions on physical activity from the WHO Survey (Q4030- Q4038). To supplement this, participants will also be asked to wear a GENEactiv accelerometer on their non-dominant wrist continuously (i.e. 24 hours a day) for 7 days. The devices were initialised before and downloaded after each use. Participants returned the device to the clinic at the DSME session at baseline. At follow-up they were given a stamped addressed envelope to mail it back to the research team.
Diet composition	Dietary habits were queried using questions from the Malawi STEPS survey (2009). Participants will be asked to report the number of days in the last week they have consumed at least one piece of fruit, at least one piece of veg, the number of fruit servings per day on an average day and the number of veg servings per day on an average day.

References

1. Polonsky WH, Anderson BJ, Lohrer PA, et al. Assessment of diabetes-related distress. *Diabetes care* 1995; **18**(6): 754-60.
2. https://www.who.int/healthinfo/survey/en/. 2004.
3. http://www.who.int/chp/steps/malawi/en/. 2009.
4. Kroenke K, Spitzer RL, Williams JB. The PHQ-9: validity of a brief depression severity measure. *Journal of general internal medicine* 2001; **16**(9): 606-13.
5. van Steenbergen-Weijenburg KM, de Vroege L, Ploeger RR, et al. Validation of the PHQ-9 as a screening instrument for depression in diabetes patients in specialized outpatient clinics. *BMC health services research* 2010; **10**: 235.
6. McGuire BE, Morrison TG, Hermanns N, et al. Short-form measures of diabetes-related emotional distress: the Problem Areas in Diabetes Scale (PAID)-5 and PAID-1. *Diabetologia* 2010; **53**(1): 66-9.
7. Stewart AL, Hays RD, Ware JE, Jr. The MOS short-form general health survey. Reliability and validity in a patient population. *Medical care* 1988; **26**(7): 724-35.
8. https://www.psykiatri-regionh.dk/who-5/Pages/default.aspx.
9. J S. Validity and reliability of the DMSES UK: a measure of self-efficacy for type 2 diabetes self-management. *Primary Health Care Research & Development* 2009: 1-8.

EXTEND Supplementary material 5: **Other medical history and family medical history**

Characteristics	Malawi<br>n = 50	Mozambique<br>n = 48	Overall<br>n = 98
**Medical history**			
Wheezing/whistling chest, n (%)	2 (4·0)	4 (8·3)	6 (6·1)
Wheezing short of breath	4 (8·0)	2 (4·2)	6 (6·1)
Asthma/bronchitis, n (%)	4 (8·0)	6 (12·5)	10 (10·2)
**Family Medical History**			
Cardiovascular disease, n (%)	0 (0·0)	7 (14·6)	7 (7·1)
Stroke, n (%)	4 (8·0)	12 (25·0)	16 (16·3)
High blood pressure, n (%)	20 (40·0)	33 (68·8)	53 (54·1)
High cholesterol, n (%)	1 (2·0)	3 (6·3)	4 (4·1)
Gestational diabetes, n (%)	1 (2·0)	0 (0·0)	1 (1·0)
Type 1 Diabetes, n (%)	7 (14·0)	0 (0·0)	7 (7·1)
Type 2 Diabetes, n (%)	17 (34·0)	28 (58·3)	45 (45·9)
Depression, n %)	0 (0·0)	4 (8·3)	4 (4·1)
Sleep Disorder, n (%)	0 (0·0)	6 (12·5)	6 (6·1)

Please note: A diagnosis of depression was not collected from participants· % percent

EXTEND Supplementary material 6: Type of medication

	Baseline			Follow-up		
**Type of Medication**	**Malawi**	**Mozambique**	**Overall**	**Malawi**	**Mozambique**	**Overall**
	n = 50	n = 48	n = 98	n = 47	n = 47	n = 94
Diet and life style only	None	None	None	None	None	None
Mono or combination therapy, n (%)						
Mono therapy						
Metformin	4 (8·0)	25 (52·1)	29 (29·6)	6 (12·8)	12 (25·5)	18 (19·1)
Sulfonylurea	1 (2·0)	5 (10·4)	6 (6·1)	0 (0·0)	3 (6·4)	3 (3·2)
Insulin	0 (0·0)	5 (10·4)	5 (5·1)	0 (0·0)	6 (12·8)	6 (6·4)
Dual therapy						
Metformin + Sulfonylurea	45 (90·0)	5 (10·4)	50 (51·0)	41 (87·2)	12 (25·5)	53 (56·4)
Metformin + Insulin	0 (0·0)	8 (16·7)	8 (8·2)	0 (0·0)	14 (29·8)	14 (14·9)
Anti-hypertension medication, n (%)	36 (72·0)	29 (60·4)	65 (66·3)	31 (62·0)	32 (66·7)	63 (67·0)
Diuretics	26 (52·0)	22 (45·8)	48 (49·0)	16 (34·0)	19 (40·4)	35 (37·2)
Calcium channel blocker	22 (44·0)	19 (39·6)	41 (41·8)	25 (53·2)	20 (42·6)	45 (47·9)
ACE-Inhibitor	8 (16·0)	16 (33·3)	24 (24·5)	3 (6·4)	15 (31·9)	18 (19·1)
Beta blocker	4 (8·0)	8 (16·7)	12 (12·2)	3 (6·4)	7 (14·9)	10 (10·6)
Centrally acting anti-hypertensive	0 (0·0)	1 (3·5)	1 (1·0)	1 (2·1)	2 (4·3)	3 (3·2)
Aspirin	1 (2·0)	0 (0·0)	1 (1·0)	1 (2·1)	0 (0·0)	1 (1·1)
Lipid lowering medication, n (%)						
Statins (Simvastatin)	0 (0·0)	13 (27·1)	13 (13·3)	0 (0·0)	12 (25·0)	12 (12·8)

Please note: Medication adherence was not collected· Data provided count (%)

Brady EM, *et al. BMJ Open* 2022; 11:e047425. doi: 10.1136/bmjopen-2020-047425
