# Supplementary material for: EXTending availability of self-management structured EducatioN programmes for people with type 2 Diabetes in low-to-middle income countries (EXTEND)—a feasibility study in Mozambique and Malawi
Source: BMJ Open. 2021 Sep 21;11(9):e047425. doi: 10.1136/bmjopen-2020-047425 (PMC8458338; doi:10.1136/bmjopen-2020-047425)
Supplement: Author's manuscript [file bmjopen-2020-047425.draft_revisions.pdf]

BMJ Open

BMJ Open is committed to open peer review. As part of this commitment we make the peer review history of every article we publish publicly available.

When an article is published we post the peer reviewers' comments and the authors' responses online. We also post the versions of the paper that were used during peer review. These are the versions that the peer review comments apply to.

The versions of the paper that follow are the versions that were submitted during the peer review process. They are not the versions of record or the final published versions. They should not be cited or distributed as the published version of this manuscript.

BMJ Open is an open access journal and the full, final, typeset and author-corrected version of record of the manuscript is available on our site with no access controls, subscription charges or pay-per-view fees (http://bmjopen.bmj.com).

If you have any questions on BMJ Open's open peer review process please email info.bmjopen@bmj.com

BMJ Open

EXTending availability of self-management structured EducatioN programmes for people with type 2 Diabetes in low-to-middle income countries (EXTEND) – A feasibility study in Mozambique and Malawi

Journal:	*BMJ Open*
Manuscript ID	bmjopen-2020-047425
Article Type:	Original research
Date Submitted by the Author:	08-Dec-2020
Complete List of Authors:	Brady, Emer M.; University Hospitals of Leicester NHS Trust, Leicester Diabetes Centre<br>Bamuya, Catherine; Malawi Epidemiology and Intervention Research Unit<br>Beran, David; Division of Tropical and Humanitarian Medicine<br>Correia, Jorge ; University Hospitals Geneva, Unit of Patient Education, Division of Endocrinology<br>Crampin, Amelia; London School of Hygiene and Tropical Medicine<br>Damasceno, Albertino; Universidade Eduardo Mondlane<br>Davies, Melanie; University of Leicester, Diabetes Research Centre<br>Hadjiconstantinou , M ; University of Leicester, Diabetes Research Centre<br>Harrington, Deirdre; University of Leicester, Diabetes Research Centre<br>Khunti, Kamlesh; University of Leicester, Diabetes Research Centre<br>Levitt, Naomi; University of Cape Town<br>Magaia, Ana; Eduardo Mondlane University, Faculty of Medicine<br>Mistry, Jayna; University Hospitals of Leicester NHS Trust, Leicester Diabetes Centre<br>Namadingo, Hazel; Malawi Epidemiology and Intervention Research<br>Rodgers, Anne; University Hospitals of Leicester NHS Trust, Leicester Diabetes Department<br>Schreder, Sally; University Hospitals of Leicester NHS Trust, Leicester Diabetes Centre<br>Simango , Leopoldo ; Mozambican Diabetes Association (AMODIA)<br>Stribling , Bernie; University Hospitals of Leicester NHS Trust, Leicester Diabetes Centre<br>Taylor, Cheryl; University Hospitals of Leicester NHS Trust, Leicester Diabetes Centre<br>Waheed, Ghazala; University of Leicester, Health Sciences
Keywords:	DIABETES & ENDOCRINOLOGY, MEDICAL EDUCATION & TRAINING, EDUCATION & TRAINING (see Medical Education & Training)

SCHOLARONE™
Manuscripts

I, the Submitting Author has the right to grant and does grant on behalf of all authors of the Work (as defined in the below author licence), an exclusive licence and/or a non-exclusive licence for contributions from authors who are: i) UK Crown employees; ii) where BMJ has agreed a CC-BY licence shall apply, and/or iii) in accordance with the terms applicable for US Federal Government officers or employees acting as part of their official duties; on a worldwide, perpetual, irrevocable, royalty-free basis to BMJ Publishing Group Ltd ("BMJ") its licensees and where the relevant Journal is co-owned by BMJ to the co-owners of the Journal, to publish the Work in this journal and any other BMJ products and to exploit all rights, as set out in our licence.

The Submitting Author accepts and understands that any supply made under these terms is made by BMJ to the Submitting Author unless you are acting as an employee on behalf of your employer or a postgraduate student of an affiliated institution which is paying any applicable article publishing charge ("APC") for Open Access articles. Where the Submitting Author wishes to make the Work available on an Open Access basis (and intends to pay the relevant APC), the terms of reuse of such Open Access shall be governed by a Creative Commons licence – details of these licences and which Creative Commons licence will apply to this Work are set out in our licence referred to above.

Other than as permitted in any relevant BMJ Author's Self Archiving Policies, I confirm this Work has not been accepted for publication elsewhere, is not being considered for publication elsewhere and does not duplicate material already published. I confirm all authors consent to publication of this Work and authorise the granting of this licence.

For peer review only

EXTending availability of self-management structured EducatioN programmes for people with type 2 Diabetes in low-to-middle income countries (EXTEND) – A feasibility study in Mozambique and Malawi

Emer M Brady PhD[1]; The EXTEND Collaborative

Catherine Bamuya MSc[2], David Beran PhD[3], Jorge C Correia MD[4], Amelia C Crampin [2], Albertino Damasceno PhD[5], Melanie J Davies MD[1,6] , Michelle Hadjiconstantinou PhD [1,6] , Deirdre M. Harrington PhD [1,6], Kamlesh Khunti MD[1,6],Naomi Levitt [7], Ana Magaia MD[5], Jayna Mistry BA[1], Hazel Namadingo MSc[2], Anne Rodgers BSc[1], Sally Schreder MSc [1], Leopoldo Simango MPH [8], Bernie Stribling MSc [1],Cheryl Taylor MSc[1], Ghazala Waheed MSc[1,6].

Affiliations:

[1] Leicester Diabetes Centre, University Hospitals of Leicester NHS Trust UK

[2] Malawi Epidemiology and Intervention Research Unit, Malawi

[3] Division of Tropical and Humanitarian Medicine, Faculty of Medicine, University of Geneva and Geneva University Hospitals, Switzerland

[4] Unit of Patient Education, Division of Endocrinology, Diabetology, Nutrition and Patient Education, WHO Collaborating Center, Department of Medicine, University of Geneva and Geneva University Hospitals, Geneva, Switzerland

[5] Faculty of Medicine, Eduardo Mondlane University, Mozambique

[6] Diabetes Research Centre, College of Life Sciences, University of Leicester, UK

[7] University of Cape Town, Cape Town, South Africa

[8] Mozambican Diabetes Association, Maputo, Mozambique

Corresponding author

Dr Emer M Brady
Cardiovascular Sciences
College of Life Sciences
University of Leicester
UK
emb29@le.ac.uk

Word count (minus abstract): 3,485

For peer review only

Abstract (244)

Background

Globally, there are estimated 425 million people with Type 2 Diabetes (T2D) globally with 80% from Low-Middle Income Countries (LMIC). Diabetes Self-Management Education (DSME) Programmes are a vital and core component of the treatment pathway for T2D. Despite LMIC being disproportionally affected by T2DN there are no DSME available that meet international diabetes federation criterion.

Methods

The aims were to test the feasibility of delivering a proven effective and cost-effective approach used in a UK population in two urban settings in Malawi and Mozambique by; 1) developing a culturally, contextually and linguistically adapted DSME, The EXTEND programme; 2) evaluating delivering training and the EXTEND programme to patients with T2D. Data collected at 0 and 6 months.

Results

Twelve healthcare professionals were trained. Ninety-eight participants received the DSME. Retention was high (100% in Mozambique and 94% in Malawi). At 6 months HbA1c (-0.9%), cholesterol (-0.3mmol/L), blood pressure (-5.9mmHg systolic and -6.1mmHg diastolic) improved in addition to indicators of wellbeing (problem areas in diabetes and self-efficacy in diabetes).

Conclusion

It is feasible to deliver and evaluate the effectiveness of a culturally, contextually and linguistically adapted EXTEND programme in two LMIC. The DSME was acceptable with positive biomedical and psychological outcomes but requires formal testing with cost-effectiveness. Challenges exist in scaling up such an approach in health systems that do not have resources to address the challenge of diabetes.

For peer review only

For peer review only - http://bmjopen.bmj.com/site/about/guidelines.xhtml

Strengths and Limitations of this study

1) This was not a randomised controlled trial but a feasibility study that included patient and patient related outcome measures
2) No control group was included in this feasibility study as it was considered unethical at this stage to deny people with Type 2 diabetes (T2D) the diabetes self-management education (DSME) programme
3) Standard operating procedures were created and utilised throughout the study for all data capture
4) Data we double entered (and discrepancies corrected with source data) from paper case report forms into a secure web-based database specifically designed for the study
5) Patients with T2D and healthcare professionals involved in delivering their care had direct involvement in the cultural and linguistic adaptation of the UK based DSME at each locality

For peer review only

For peer review only - http://bmjopen.bmj.com/site/about/guidelines.xhtml

Introduction

The global estimates of prevalent cases of Type 2 Diabetes (T2D) are approximately 500 million [1], with a further 212 million thought to be undiagnosed [2] with a higher proportion seen in Low-middle income-countries (LMIC) [3] . Poor glycaemic control, the hallmark of T2D, accounts for 10.7% of global all-cause mortality in people aged between 20-79 years old [2].

T2D is a progressive chronic condition, when sub-optimally managed, can lead to the development of both microvascular and macrovascular complications, including for example retinopathy, nephropathy, and neuropathy, heart disease, peripheral vascular disease, resulting in end organ damage in approximately one third to one half of people with T2D [4]. This is concerning particularly in those health systems, such as in LMICs, that are fragmented, overstretched and under resourced, with intermittent drug supplies and rudimentary clinical training on T2D.

The vast economic burden of T2D includes both direct costs from medical care and indirect costs via loss of productivity or earnings, summing to some estimated $1.3 trillion [5]. It is estimated that the healthcare costs for a person with diabetes is two-fold higher than without diabetes. This global public health issue is impacting the world's poorest countries; indeed 80% of all cases of T2D come from LMIC such as those within Sub-Saharan Africa (SSA). The current epidemiological transition occurring in SSA, due to rapid urbanisation and nutritional shift, has seen an increase in the burden of T2D [2]. It is predicted that by 2045, 47.1 million people in SSA will have T2D [6]. The additional and increasing strain of T2D placed on their already stretched health systems highlights the need to find cost-effective approaches to reducing the disease burden.

Leading international health organisations promote self-management structured education as the cornerstone of diabetes care, and recommend Diabetes Self-Management Education (DSME) programmes as a core component of the treatment pathway for diabetes [7-9]. The overall objectives of

For peer review only - http://bmjopen.bmj.com/site/about/guidelines.xhtml

DSME are to support informed decision-making, self-care behaviours, problem-solving and active collaboration with the health care team and to improve clinical outcomes, health status, and quality of life [10]. DSME offer a potential financially viable treatment option for healthcare settings within both high-income countries (HIC) and LMIC.

A recent systematic review highlighted that despite there being a number of studies looking at self-management behaviours in SSA, self-management itself is insufficient in these countries. In particular patients do not engage, or are aware in some cases, of risk reducing behaviours such as physical activity, reducing salt-intake and good foot care [11]. Furthermore, the DSME described in these studies do not meet the standards set-out by the National Institute of Clinical Excellence (NICE) in the UK, that is, that they include certain components [8] for example an evidence-base, suits the needs of the person, has specific learning objectives, that supports the person in developing attitudes, beliefs, knowledge and skills to self-manage diabetes; have a structured curriculum that is theory-driven, evidence-based and resource-effective, with supporting materials, and is written down; Delivered by trained educators; is quality assured and so on. These principals are supported by the American Diabetes Association, European Association for the Study of Diabetes [7] and the International Diabetes Federation [12]. To our knowledge there are no DSME programmes with proven effectiveness and cost-effectiveness in SSA that meet these criteria.

The aim of this study was to take a culturally and contextually adapted DSME programme, the EXTEND programme, that meets criteria for DSME and has proven cost-effectiveness and effectiveness in a UK population [13-15], and test the feasibility of working with local teams to deliver training, recruiting patients, delivering the programme and collecting biomedical and psychological research outcomes in two SSA urban settings in Malawi (Lilongwe) and Mozambique (Maputo).

For peer review only

For peer review only - http://bmjopen.bmj.com/site/about/guidelines.xhtml

Methods

Ethical approval was granted by: the Scientific Commission of the Faculty of Medicine and the Mozambique National Research Ethics Committee (CNBS – Comité National de Bioética para Saúde); the College of Medicine Research Ethics Committee, Malawi; and the University of Leicester College of Life Sciences research ethics committee. This study was funded by the Global Challenges Research Fund NCDs Foundation Awards 2016 Developmental Pathway Funding (Medical Research Council). All participants were offered the intervention. Data were collected after informed consent was obtained and before the intervention (baseline) and at 6 months.

Participants

Patients were recruited from the Mozambican Diabetes Association (AMODIA) out-patients' clinic, in Maputo. In Malawi patients were identified from the health centre in Area 25 in Lilongwe. Eligibility were a diagnosis of T2D and 18 years old or older. Exclusion criteria were: severe and enduring mental health problems; not primarily responsible for their own care, could not provide informed consent; not able to participate in activities in a group setting; or currently participating in another intervention study.

Sample size

No formal sample size was calculated because this is a feasibility study. A sample size of 50 participants in each site was selected based on a balance between pragmatism and having a large enough sample to produce reasonable parameter estimates to power a future formal evaluation of the EXTEND programme and experience of the logistics of its delivery.

The intervention

The DSME programme, named "EXTEND", aimed to extend the availability of self-management structured education programme for people with T2D in LMIC. EXTEND is an interactive group based

For peer review only - http://bmjopen.bmj.com/site/about/guidelines.xhtml

programme culturally and contextually adapted from a programme first developed and tested in the UK [13-15] and meets international guidelines for DSME. It was delivered in two 3-hour sessions by two trained educators to people with T2D in Portuguese in Maputo and Chicheŵa in Lilongwe.

Patient and Public Involvement

Patient and public involvement was of critical importance during the adaptation of the UK based DSME programme. The EXTEND programme was co-produced by the EXTNED investigators and patients, educators, nurses and patients spouses/children. The UK DSME with supporting resources were taken to Mozambique and Malawi separately by the national educators. At site the local research teams had invited patients and those who would eventually be trained to deliver the education to be part of the PPI group and attend a two-day session where all content were shared and scrutinised by the local PPI group. Sections of the DSME that were not relevant to the local population were removed or amended. Local foods and cooking practices were included as directed by the PPI groups and other topics of importance for example erectile dysfunction and natural remedies for diabetes. The adaptations were made back in the UK in collaboratively with the local researchers. The DSME was then translated which prompted further amendments given differences on vernacular. The UK national educators then returned the each locality and delivered EXTEND to two new groups of patients and their spouse/child and received further alterations. During the delivery of EXTEND in the study further suggestions made during the study were collected and feedback and incorporated into the final version.

Educator training

Educators who delivered EXTEND were trained by accredited national educator trainers from the UK who conducted two-day training sessions in each of the settings; a total of eight educators were successfully trained in Lilongwe and four in Maputo. The trainers' set-up a WhatsApp group with each group of educators to provide on-going support remotely as requested by the educators. In Malawi

For peer review only - http://bmjopen.bmj.com/site/about/guidelines.xhtml

the people trained consisted of; 3 nurses and 5 lay people with T2D. The research associate at location selected 4 of the most competent educators to take part in the feasibility study which included 2 nurses and 2 lay people with T2D. The remaining 4 went back into their communities to spread the messages. In Mozambique the people trained included 3 nurses and a medical student all 4 took part in the feasibility study.

Participants and educators were provided refreshments and refunded travel expenses at both baseline, education and follow-up visits.

Feasibility related outcomes

Feasibility related outcomes were collected via a recruitment log completed by the onsite recruitment team:

1. Number of eligible patients referred who accepted the invitation and number who refused
2. Number of eligible patients referred who accepted the feasibility study invitation and attended DSME and research study visits (baseline only, baseline and follow-up)
3. Data collected at each visit
4. Baseline characteristics of the sample who were enrolled in the study
5. Retention rate
6. Change values for each of the potential outcome measures

Participant outcome data

Participants that consented to the feasibility study had the baseline data collected before EXTEND was delivered and at 6 months. Data were recorded in a paper-based case report form (CRF). Standard operating procedures (SOPs) were developed, agreed and followed at both sites. Data were double entered into a REDCap [16] database, which uses a 'My Structured Query Language' (MySQL) database (an open-source relational database management system (RDBMS)) via a secure web interface, with

For peer review only - http://bmjopen.bmj.com/site/about/guidelines.xhtml

data checks used during data entry to ensure quality. All supporting systems were hosted and housed within the secure networked environment provided by the University of Leicester, UK.

Data collected are provided in *supplementary material 1* but in summary include: demographics, medical history, anthropometric measurements, cardio-metabolic related outcomes, psychological outcomes pertaining to health and wellbeing and lifestyle behaviours including objectively measured physical activity.

Qualitative study

The qualitative study (focus group discussions) was conducted in both settings with the purpose of exploring the views and experiences of those directly involved with the EXTEND programme (i.e. people with T2D, trainers, educators). In addition, the views of clinicians and stakeholders who are regularly in contact with people with T2D were explored on the potential for future implementation. In depth results are presented elsewhere. Here, an overview of the participants' views on and experience with the EXTEND programme is provided.

For peer review only

Analysis

Quantitative data analysis

Descriptive statistics were produced for participant characteristics at baseline using mean (SD) for normally distributed variables, median (IQR) for non-normally distributed variables, count and percentage (%) for categorical variables. Each of the outcome measure has been summarised using appropriate descriptive statistics at baseline and at 6 months. A paired t test for normally distributed continuous variables (or Mcnemar's test for categorical variables) was used to compare baseline and post intervention means (or proportions) separately in each country. Non-parametric Wilcoxon ranksum test was used to compare baseline and follow-up medians when the data was not normally distributed.

For peer review only - http://bmjopen.bmj.com/site/about/guidelines.xhtml

Qualitative data analysis

Taking an inductive thematic approach, data was analysed by two researchers (MH and CB) based on the Framework method [17] and applying principles of the constant comparative techniques [18]. Data was organised with the use of the NVivo qualitative data indexing software.

Results

Recruitment and retention

A total of 98 participants, 50 in Malawi and 48 in Mozambique were recruited to the feasibility study. Overall the mean age was 55·2 years and 62% were female. The retention rate was high (Figure 1). All outcome data were successfully collected in both sites for both study time-points with the exception of the objective measure of physical activity, which was only collected in Malawi (at baseline). In pairs the educators delivered EXTEND to all participants.

Baseline characteristics (Table 1)

Twenty-two per cent of participants were within the accepted “normal” range for BMI, 35% were overweight and 43% as obese. Overall, between 60-78% had hypertension with a low prevalence of hypercholesterolemia in Malawi compared to 41% of the Mozambican cohort. A family history of T2D and/or hypertension was common (supplementary material 2). HbA1c was similar between the two cohorts and indicative of poor glycaemic control. Fasting glucose was higher in Mozambique but in both settings was > 7·0mmol/L, again indicating sub-optimal control. Despite total cholesterol levels being within the ‘healthy’ range HDLc was low “normal” and LDLc was high “normal” which are considered to be associated with increased risk of heart disease. Current medication is provided in supplementary material 3; no participants were managing their T2D with diet and lifestyle only. The majority were on dual therapy of metformin and sulphonylurea and none on insulin therapy at the time of the study in Malawi. In Mozambique the majority were using monotherapy (>70%). Over 60% in both cohorts were taking anti-hypertensive medication and a quarter of those in Mozambique were

For peer review only - http://bmjopen.bmj.com/site/about/guidelines.xhtml

on lipid-lowering medication. No participants in Malawi reported being prescribed lipid lowering medication. Data were not collected on medicine adherence. There was a shift from monotherapy (metformin) to dual therapy from baseline to follow-up; metformin plus sulphonylurea increased by 5% and metformin plus insulin by 7%. There was a small reduction in those receiving a diuretic at six months.

For peer review only

For peer review only - http://bmjopen.bmj.com/site/about/guidelines.xhtml

Table 1| Baseline characteristics

	Malawi n = 50	Mozambique n = 48	Overall n = 98
**Demographics**			
Age	56·2 (11·6)	54·2 (7·8)	55·2 (9·9)
Gender (female)	30 (60·0)	31 (64·6)	61 (62·2)
Duration T2DM (years)	6·8 (5·5)	8·81(5·9)	7·79(5·8)
**Medical History (n, %)**			
Hypertension	39 (78·0)	29 (60·4)	68 (69·4)
High cholesterol	2 (4·0)	20 (41·7)	22 (22·5)
Last time cholesterol checked (months)*	0 (0 – 9)	1 (1 – 2)	1 (0 – 3)
Stroke	5 (10·0)	1 (2·1)	6 (6·1)
Heart disease	0 (0·0)	1 (2·1)	1 (1·0)
Tuberculosis	4 (8·0)	4 (8·3)	8 (8·2)
**Biomedical characteristics**			
HbA1c (%)*	9·7 [7·9 –14·7]	9·6 [7·6 –14·7]	9·6[7·7– 14·7]
HbA1c (mmol/mol)*	102·6 (48·4)	95·1 (46·5)	98·9 (47·4)
Fasting glucose (mmol/L)*	7·5 [5·5 –10·3]	9·0 [6·5 –13·2]	8·1[6·2– 12·0]
Total cholesterol (mmol/L) **	5·1 (1·3)	4·7 (0·9)	4·9 (1·2)
HDL (mmol/L)**	1·3 (0·3)	1·2 (0·3)	1·2 (0·3)
LDL (mmol/L) **	3·4 (1·0)	2·8 (0·9)	3·1 (1·0)
Triglycerides (mmol/L)*	1·5 [1·0 – 1·9]	1·1 [0·8 – 1·6]	1·3[0·9-1·8]
Systolic BP (mmHg) **	136·8 (20·6)	142·8 (22·6)	139·7 (21·7)
Diastolic BP (mmHg)**	86·8 (8·9)	89·6 (10·8)	88·2 (9·9)
Weight (Kg) **	71·2 (13·9)	82·2 (17·8)	76·5 (16·7)
BMI (Kg/m^2) **	27·4 (5·3)	29·5 (6·4)	28·5 (6·0)
**BMI categories**			
Normal (20-24·9 Kg/m^2)**	11 (23·4)	10 (21·3)	21 (22·3)
Overweight (25-29·9 Kg/m^2)**	19 (40·4)	14 (29·8)	33 (35·1)
Obese (≥ 30 Kg/m^2)**	17 (36·2)	23 (48·9)	40 (42·6)
Waist circumference (cm)**	91·8 (12·6)	95·6 (15·5)	93·6 (14·2)

* Median [Interquartile range [IQR]] for non-normally distributed variables, ** Mean (SD) for normally distributed variables.
HbA1c: Glycated haemoglobin; HDL: High Density Lipoprotein; LDL: Low Density Lipoprotein; BMI: Body Mass Index

For peer review only

Changes in biomedical and psychological outcomes from baseline (Table 2 and 3, respectively)

Although, this feasibility study was not powered to detect statistically significant differences in biomedical outcomes, overall the reductions in HbA1c (-0·9% (95%CI: -1·4 to-0·1)), total cholesterol (-0·3mmol/L (95%CI: -0·4 to -0·1)), LDL cholesterol (-0.2mmol/L (95%CI: -0·3 to -0·0)), triglycerides (-0.2mmol/L(95%CI: -0·3 to -0·1)), diastolic (-6.1mmHg (95%CI: -8·2 to -4·0)) and systolic (-5·9mmHg, (95%CI: -9·6 to -2·1)) blood pressure are clinically important and do reach statistical significance. Heart rate, weight and BMI increased negligibly in both cohorts. A clinically relevant reduction in HbA1c, glucose and diastolic blood pressure are observed in participants in Malawi with modest reductions also observed for total cholesterol, LDL cholesterol and weight at six months follow-up. The same pattern is seen in participants from Mozambique in addition to a significant reduction in systolic blood pressure.

Overall the prevalence of 'non-minimal presence of depressive symptoms' (as measured by PHQ-9) at baseline was high at 52% and increased at six months. There were no reported cases of severe depressive symptoms in either cohort (score 20-27). Malawi and Mozambique had similar levels of mild and moderate depression at baseline (Malawi 32% and 10% respectively, Mozambique 39.6% and 10.4%, respectively) with a lower prevalence for both categories at 6 months. The Problem Areas in Diabetes (PAID) showed a significant improvement with an overall reduction of approximately 7 points at six months i.e. a lower score is indicative of fewer problems associated with diabetes. A comparable reduction was observed between the two time-points in each setting (Malawi 5 points and Mozambique approximately 9 points). Overall wellbeing (WHO (Five)) was high in both settings and remained essentially unchanged in Malawi but there was a reduction in the proportion of those likely to have depression (score <25) in Mozambique at 6 months. Overall self-efficacy for diabetes improved at six months reaching statistical significance. Results from the 20-Item Short Form Health Survey (SF-20) indicated a high and maintained level of physical, role and social functioning. In addition

For peer review only - http://bmjopen.bmj.com/site/about/guidelines.xhtml

a reduction in pain and statistically significant improvement in health perceptions at six months was observed.

For peer review only

For peer review only - http://bmjopen.bmj.com/site/about/guidelines.xhtml

Table 2 | Mean change in biomedical characteristics from baseline

	Malawi				Mozambique				Overall			
	**Baseline**	**Follow-up**	**Mean change (95% CI) n = 47**	**P - value**	**Baseline**	**Follow-up**	**Mean change (95%CI) n = 48**	**P-value**	**Baseline**	**Follow-up**	**Mean change (95%CI) n = 95**	P-value
**Characteristic**												
HbA1c (%) **	9·7[7·9-14·7]	9·8[7·6-13·3]	-0·8 (-1·3 to 0·1)	0·058	9·6[7·6-14·7]	8·4[7·2-10·7]	-1·1(-2·5 to 0·1)	0·144	9·6[7·7-14·7]	8·8[7·4-12·1]	-0·9(-1·4 to-0·1)	0·001
HbA1c (mmol/mol)	102·6 (48·4)	89·5 (39·5)	9·7(32·2)		95·1 (46·5)	77·3(31·1)	17·8 (39·3)		98·9 (47·4)	83·4(35·8)	13·8 (35·9)	
Glucose (mmol/L)**	7·5[5·5-10·3]	7·6[5·6-10·7]	-0·4 (-0·9 to 0·4)	0·589	9·0[6·5-13·2]	7·7[5·8-12·4]	-0·9 (-1·9 to 0·4)	0·372	8·1[6·2-12·0]	7·6[5·7-12·1]	-0·4(-1·1 to 0·03)	0·313
Total cholesterol (mmol/L)	5·1 (1·3)	[2·3 – 8·2] 4·9 (1·2)	-0·2(-0·4 to -0·0)	0·046	4·7 (0·9)	4·4(1·0)	-0·3(-0·6 to -0·1)	0·016	4·9 (1·2)	4·6(1·1)	-0·3(-0·4 to -0·1)	0·002
HDL (mmol/L)	1·3 (0·3)	1·3 (0·3)	-0·1(-0·1 to 0·0)	0·069	1·2 (0·3)	1·3(0·4)	0·1(-0·0 to 0·1)	0·087	1·2 (0·3)	1·3 (0·3)	0·0(-0·1 to 0·1)	0·997
LDL (mmol/L)	3·4 (1·0)	3·3(0·9)	-0·2(-0·3 to-0·0)	0·017	2·8 (0·9)	2·6 (0·8)	-0·2 (-0·4 to 0·1)	0·147	3·1 (1·0)	2·9 (0·9)	-0·2(-0·3 to -0·0)	0·013
Triglycerides (mmol/L)**	1·5 [1 – 1·9]	1·3[0·9 -2·0]	-0·2(-0·4 to -0·1)	0·008	1·1[0·8 - 1·6]	1·0[0·8 -1·3]	-0·1(-0·3 to 0·1)	0·122	1·3 [0·9 – 1·8]	1·2 [0·8-1·7]	-0·2(-0·3 to -0·1)	0·002
Systolic BP (mmHg)	136·8 (20·6)	138·5(19·3)	0·7(-3·4 to 4·8)	0·736	142·8 (22·6)	130·4(18·9)	-12·3(-18·2to -6·5)	0·000	139·7 (21·7)	134·4(19·4)	-5·9(-9·6 to -2·1)	0·003
Diastolic BP (mmHg)	86·8 (8·9)	80·8(8·9)	-6·1(-8·9 to -3·3)	0·000	89·6 (10·8)	83·5(9·2)	-6·1(-9·4 to -2·8)	0·001	88·2 (9·9)	82·2 (9·1)	-6·1(-8·2 to -4·0)	<0·001
Heart rate (bpm)	79·7 (14·5)	80·9 (12·8)	1·3(-2·1 to 4·7)	0·437	74·3 (12·4)	76·3(10·6)	1·9 (-1·5 to 5·4)	0·269	77·1 (13·7)	78·6(11·9)	1·6(-0·8 to 4·0)	0·178
Weight (Kg)	71·1(13·9)	71·0(12·4)	0·1(-0·9 to 1·1)	0·809	[53·0 -142·0] 82·2 (17·8)	82·0 (17·2)	-0·2(-1·3 to 1·0)	0·781	76·5 (16·7)	76·5(15·9)	-0·0(-0·7 to 0·7)	0·595
Waist (cm)	91·8 (12·6)	91·0 (11·7)	-0·9 (-2·8 to 0·9)	0·323	95·6 (15·5)	98·0 (14·4)	2·4 (-0·2 to 4·9)	0·067	93·6 (14·2)	94·5 (13·5)	0·8 (-0·8 to 2·4)	0·345
BMI (Kg/m^2)	27·4 (5·4)	27·5(4·9)	0·03(-0·3 to 0·4)	0·880	29·6 (6·4)	29·5 (6·2)	-0·1(-0·5 to 0·3)	0·747	28·5 (6·0)	28·5 (5·7)	-0·0(-0·3 to 0·3)	0·892

All values are mean (Standard deviation (SD) for normally distributed variables), unless otherwise stated **Median change [range] for non-normally distributed variables

HbA1c: Glycated haemoglobin; HDL: High Density Lipoprotein; LDL: Low Density Lipoprotein; BMI: Body Mass Index

For peer review only - http://bmjopen.bmj.com/site/about/guidelines.xhtml

Table 3 | Scores for measures from questionnaire at baseline and follow-up

Questionnaire Score	Malawi Baseline N = 50	Malawi Follow-up N = 47	P-value*	Mozambique Baseline N = 48	Mozambique Follow-up N = 48	P-value*	Overall Baseline N = 98	Overall Follow-up N = 95	P-value*
Health and Wellbeing related outcomes (PHQ-9) [a]									
Depressive symptoms severity									
None-minimal (0-4), n (%)	27 (54·0)	33 (70·2)	0·134	24 (50·0)	29 (60·4)	0·297	51 (52·0)	62 (65·3)	0·078
Mild (5-9), n (%)	16 (32·0)	11 (23·4)	0·317	19 (39·6)	15 (31·3)	0·414	35 (35·7)	26 (27·4)	0·206
Moderate (10-14), n (%)	5 (10·0)	3 (6·4)	1·000	5 (10·4)	3 (6·3)	0·480	10 (10·2)	6 (6·3)	0·527
Moderately severe (15-19), n (%)	2 (4·0)	0 (0·0)	0·157	0 (0·0)	1 (2·1)	0·317	2 (2·0)	1 (1·1)	0·564
Severe (20-27), n (%)	0 (0·0)	0 (0·0)	-	0 (0·0)	0 (0·0)	-	0 (0·0)	0 (0·0)	-
Proportion with score > 10, n (%)	6 (12·0)	1 (2·1)	0·059	2 (4·2)	3 (6·3)	0·564	8 (8·2)	4 (4·2)	0·206
Proportion with score ≥ 10, n (%)	7 (14·0)	3 (6·0)	0·103	5 (10·4)	4 (8·3)	0·706	12 (12·2)	7 (7·1)	0·166
Problem Areas in Diabetes(PAID) [b]									
Raw score, Mean (SD)	11·2 (11·9)	6·0 (8·3)	0·012	21·1 (11·7)	11·5 (8·6)	<0·001	16·1 (12·7)	8·8 (8·8)	<0·001
Proportion with raw score ≥ 40, n (%)	2 (4·0)	1 (2·0)	0·564	5 (10·4)	0 (0·0)	0·025	7 (7·1)	1 (1·0)	0·034
Percentage score, Mean (SD)	14·0 (14·9)	7·6 (10·3)	0·012	26·4 (14·6)	14·3 (10·7)	<0·001	20·1 (15·9)	11·0 (11·0)	<0·001
WHO (Five) Well-Being Index [c]									
Raw score, Mean (SD)	19·1 (5·2)	20·0 (5·1)	0·514	17·6 (4·7)	16·1 (4·1)	0·049	18·4 (5·0)	18·0 (5·0)	0·382
Proportion with raw score < 13, n (%) Poor well being	6 (12·0)	4 (8·0)	0·739	7 (14·6)	10 (20·8)	0·366	13 (13·3)	14 (14·3)	0·655
Percentage score, Mean (SD)	76·5 (20·8)	80·1 (20·5)	0·514	70·6 (18·6)	64·4 (16·6)	0·049	73·6 (19·9)	72·2 (20·1)	0·382
Proportion with percentage score<25, n (%) Likely depression	1 (2·0)	1 (2·0)	0·317	4 (8·3)	0 (0·0)	0·046	5 (5·1)	1 (1·0)	0·180
Self-Efficacy for Diabetes (DSEQ) [d], Mean (SD)	68·7 (16·9)	79·8 (10·2)	0·001	70·7 (12·9)	69·1 (10·0)	0·449	69·7 (15·0)	74·4 (11·4)	0·027
**Questionnaire Score**	**Malawi Baseline** N = 50	**Malawi Follow-up** N = 47	P-value*	**Mozambique Baseline** N = 48	**Mozambique Follow-up** N = 48	P-value*	**Overall Baseline** N = 98	**Overall Follow-up** N = 95	P-value*
Medical Outcomes Study: (SF-20) [e]									
Functioning									
Physical functioning									
Median (IQR)	91·6(75-100)	100(91·6-100)	0·545	100(100-100)	100(95·8-100)	0·009	100(83·3-100)	100(91·6-100)	0·282
Role functioning									
Median (IQR)	100(50-100)	100(100-100)	0·084	100(100-100)	100(100-100)	0·183	100(100-100)	100(100-100)	0·030
Social functioning									

For peer review only - http://bmjopen.bmj.com/site/about/guidelines.xhtml

Questionnaire Score	Malawi Baseline N = 50	Malawi Follow-up N = 47	P-value*	Mozambique Baseline N = 48	Mozambique Follow-up N = 48	P-value*	Overall Baseline N = 98	Overall Follow-up N = 95	P-value*
Median (IQR)	100(100-100)	100 (100-100)	0·813	100(100-100)	100 (100-100)	0·542	100 (100-100)	100 (100-100)	0·793
Well-Being									
Mental health									
Median (IQR)	88 (76-96)	92 (84-100)	0·069	80 (68-88)	76 (64-84)	0·681	84 (72-88)	84 (72-92)	0·344
Health perceptions									
Median (IQR)	46·8(32·2-60)	65 (41·8-90)	0·000	53·6 (30 -67·2)	48·6 (32·2-61·1)	0·894	46·8 (30-62·2)	56·8(36·8-76·8)	0·005
Pain									
Median (IQR)	40 (0-60)	20 (0-60)	0·284	40 (0-60)	40 (20-60)	0·407	40 (0-60)	20 (0-60)	0·171

* P-value calculated using McNemar's test or paired ttest or non-parametric Wilcoxon ranksum test

a PHQ-9; responses were for over the last two weeks; scale for all responses ranges from 0 = Not at all to 3=Nearly every day; total scores range from 0 to 27 with higher scores indicating poor health status.

b PAID: Problem Areas in Diabetes; scale for all responses ranges from 0 = not a problem to 4 = serious problem; total scores range from 0 to 80· The score is multiplied by 1·25 to get the percentage score and a score of >=40 is severe diabetes distress

c WHO Well-Being Index; responses were for over the last two weeks; scale for all responses ranges from 0=At no time to 5=All of the time; total raw scores range from 0 to 25, 0 representing worst possible and 25 representing best possible quality of life· The raw score is multiplied by 4 to obtain a percentage score ranging from 0 to 100.

d Self-Efficacy for Diabetes (Modified version of DSEQ); scale for all responses ranges from 1 = Not at all confident to 10 = Totally confident; total scores range from 9 to 90 with higher scores indicating higher self-efficacy.

e SF-20; Physical functioning total raw score=18, Role functioning total raw score=6, Social functioning total raw score=6, Mental health total raw score=30, Health perception total raw score=25, Pain total raw score=6. All parameters scaled 0-100· A higher score indicates better functioning or well-being. The only exception is pain: a higher score indicated more pain.

nb:Mean (SD) expressed for normally distributed variables and median (IQR) for non-normally distributed variables.

For peer review only

Qualitative findings (Table 4)

Overall, participants who took part shared that the EXTEND programme had a significant positive impact on their behaviour, indicating improvements in lifestyle habits, including increasing physical activity, and improving food choices. They also reported improvements regarding losing weight and taking medication as advised by their doctor (Table 4). Participants on a whole expressed positive experience attending a DSME programme such as EXTEND and emphasised a strong need for such self-management education in their local communities. Due to lack of information and education about the management of T2D, people with this condition are often misinformed. In addition to the inconsistent information provided to people with T2D, they are also exposed to confusing and conflicting advice from clinicians highlighting that education in diabetes should not only be available for patients, but for healthcare professionals also a detailed analysis of this qualitative study is reported elsewhere.

For peer review only - http://bmjopen.bmj.com/site/about/guidelines.xhtml

Table 4: Qualitative data from patients who received the DSME

Behaviour Change	Patient Perspective
Improvements in taking medication	*I am a living example when I was first diagnosed with T2D I was prescribed to take metformin in the morning and evening but after learning about my condition especially diet topic I was able to manage my diet and as a result I was asked to take metformin once a day and as of now I am only taking half a tablet in the morning and another half in the evening (P5, non-participant, Malawi)*
Increasing physical activity	*For me, this training program was a privilege, and I've been capitalizing on everything I've learned. Especially in the alignment between eating and physical exercise, because this is where I had many problems (P4, patient, Moz)*
	I could walk from home going to Msungwi but when I reach there I could feel so much pain as if something bad is happening in my body. But after getting the education I have been walking long distances during the evening and I have seen that its working (P5, female patient, Malawi)
	The other part which I also liked most was the advice we get from the clinic such as doing different physical exercises like moving a wheel bar, cultivating the garden, so this is helping our bodies to be strong (P6, male patient, Malawi)
Improving food choices	*We use to abuse on the oil, tomato, onion, everything· Now I know how to do things moderately· I learned a lot, the course was valuable (P4, patient, Moz)*
	For me I think the program was good because they taught us how to take care of our bodies and the need to consume food that has less cholesterol (P2, female patient Malawi)
	We were just ignorant especially on the issue of diet and this was not helping us, but now after getting the education we are able to take care of ourselves (P2, female patient Malawi)
	Mmm, and the foods we were taught to eat it's our locally Malawian foods; they didn't tell us to take foreign food which we could spend thousands of money to buy, no so the examples were relevant (P2, female patient Malawi)
Manage stress	*We stopped having stress and we accepted that we are T2D patients. You also taught us how we can live long by doing exercise, having good diet and etc. The main thing is to accept and avoid stress (P5, male patient, Malawi)*
Losing Weight	*I was weighing over 105kgs but now I have reduced the weight to 92 (P6, female patient Malawi)*
	I was weighing 85kgs but am weighing 75 kgs and I feel very light now (P1, male patient Malawi)
	I already lost 4 kg. My blood sugar levels have already dropped. The tiredness I used to feel I no longer feel (P1, patient, Moz)

Data sharing statement

There are no other data available

<u>Discussion</u>

This study demonstrates that it is possible and acceptable to deliver the culturally, contextually and linguistically adapted EXTEND programme in an urban setting in two LMIC. A study to recruit to and collect data for the purpose of evaluating the biomedical and psychological impact of the EXTEND programme was successfully developed and implemented.

The incomplete objective physical activity data both sites means that future collection will require more support. Objective measures of physical activity is important in LMIC and NCD populations because there is a dearth of evidence in this area. A number of additional important data points should

For peer review only - http://bmjopen.bmj.com/site/about/guidelines.xhtml

be included in future studies including; duration of diabetes, age at diagnosis, history of depression, medicine adherence, access to medicine, previous education in diabetes, communicable disease comorbidities i.e. HIV/AIDS .

It was anticipated that the development of the protocol would take circa 12 weeks however it took 26 weeks therefore a longer time-frame for the development of these core documents should be built into any future work. Due to the large interest and requests from people with T2D for the DSME programme (after recruitment had ceased) as reported by local research teams it is preferable that a future effectiveness study focuses on less traditional study designs, for example step-wedged or wait lists, or build in infrastructure/finance to permit control arm participants to receive the programme at the end of the study at no personal cost to them.

The baseline data indicated people with T2D in the two urban settings have poorer glycaemic control than their UK counterparts [19] and by comparison to the pooled baseline HbA1c, from a systematic review conducted by Steinsbekk and colleagues in 2012, that included individuals from the USA, Sweden and Thailand pre- and post DSME [20]. It is well recognised that health outcomes for patients with T2D are largely dependent on the individual's ability to effectively implement and sustain complex self-management skills into their daily lives [20 21]. Encouragingly, whilst this feasibility study was not powered to detect a change in outcomes at 6 months both clinically and statistically significant changes in biomedical and psychological outcomes are observed. The reduction in HbA1c, for example, that is approaching 1% is clinically meaningful for example a 1% reduction in HbA1c reportedly reduces the risk for any end point related to diabetes by 21% [22]. Further, reductions in SBP and Diastolic Blood Pressure (DBP) of ≥2 mm Hg are reported to significantly reduce the incidence of CVD [23 24], thus the reduction of -5·9 and -6·1 (for SBP and DPB, respectively) can be considered clinically meaningful. These data are in-line with evidence from previously tested DSME programmes in HIC [20]. Our data also indicate that these changes are not driven by weight loss as one might expect but may

For peer review only - http://bmjopen.bmj.com/site/about/guidelines.xhtml

be instead attributed to the reported lifestyle changes (Table 4) and a greater understanding of their condition and thus adherence to both glucose lowering and anti-hypertensive medications.

The improvements observed in self-efficacy and knowledge of diabetes are supported by previously reported DSME [25 26] and is the core for successful self-management. Behaviour change is not determined solely by knowledge and information, it is however, fundamental that the individual understands their condition and is equipped with the appropriate skills and confidence to self-manage. The improved diabetes distress score suggests that attending a self-management programme could have a positive impact on behaviour change and emotional wellbeing (PAIDS) [27]. The data from the qualitative study support the acceptability and need for a DSME such as EXTEND in LMICs, given the lack of available education around T2D and their limited access to support for the management of their diabetes.

The rising prevalence of T2D in LMICs, whose healthcare systems are already under immense pressure with infectious disease and limited resources, highlights the need for low-cost effective interventions. This feasibility study has demonstrated the short-term benefits of the DSME EXTEND that meets international principles for self-management education. A definitive trial that includes multiple settings (urban, rural and remote) and cost outcomes is required to formally evaluate the effectiveness and cost-effectiveness of this DSME, and be powered to examine impact on clinical outcomes, diabetes complications with adequate follow-up to explore the persistence of any changes observed in outcome measures. It should also address the sustainability of such programmes in the settings they are tested with implementation pathways and buy-in from influential stakeholders and national decision makers from the off-set. It is advisable to employ the 'train the trainer' model and use lay educators which has been shown to be as effective as HCP provision in the UK [28].

Study limitations include it was only delivered in urban settings thus results are not generalizable to the wider diabetes populations e.g. rural and remote dwellers. Study strengths include use of standardised case report forms (CRF) and standard operating procedures at each site, double entry of

For peer review only - http://bmjopen.bmj.com/site/about/guidelines.xhtml

all data, the successful recruitment of desired sample and high retention rate. All data were collected at each site for both time-points except objective measure of physical activity. Lastly the significant and clinically relevant reduction in biomedical, psychological parameters and the patient experience demonstrate a need for DSMEs such as EXTEND.

Conclusion

It is feasible to train educators to successfully deliver a fit-for-purpose DSME in urban settings in two LMIC. The positive biomedical and psychosocial outcomes observed warrant the formal evaluation of the effectiveness, cost-effectiveness and sustainability of the EXTEND programme in Malawi and Mozambique.

Funding Statement

This work was funded by the Medical Research Council Global Challenges Research Fund NCDs Foundation Awards 2016 Developmental Pathway.

Author's contribution

EMB: Study design. Protocol, case report form and database design. Data cleaning, analysis and interpretation. Development of intervention. Writing and revision of manuscript, figures and tables. CB and AM: Data acquisition, entry and cleaning. LS:Delivery of intervention. Data acquisition, entry and cleaning. Revision of manuscript. JM: Development of intervention. Database design. Data cleaning. Revision of manuscript. MJD: Study and protocol design. Development of intervention. Data analysis and interpretation. Revision of manuscript.AD: Study and protocol design. Case report form design. Data interpretation. Revision of manuscript. SS, BS, CT and AR: Development and delivery of intervention. Data interpretation. Revision of manuscript. MC: Study and protocol design. Development of intervention. Case report form and database design Data interpretation. Revision of manuscript. HN: Protocol and case report form design. Development of intervention. Revision of manuscript. DB, JCC, MH, DMH, KK and NL: Study and protocol design. Data interpretation. Revision of manuscript. GW: Statistical analysis. Revision of manuscript.

Competing Interests

KK reports grants and or personal fees from Amgen, Astrazeneca, Baye, NAPP, Lilly, Merck Sharp and Dohme, Novartis, Novo Nordisk, Roche, Berlin-Chemie AG/Menarini Group, Sanofi-Aventis, Servier, Bohringer Ingelheim

For peer review only - http://bmjopen.bmj.com/site/about/guidelines.xhtml

and Pfizer outside the submitted work. MJD reports grants from University of Leicester and international development fund during this work. DH reports non-financial support from Novo Nordisk during this work. No other author has any declarations.

Acknowledgments

Individual REDCap study databases and ongoing data management were developed and conducted in partnership with the NIHR Leicester Biomedical Research Centre Bioinformatics Hub.

For peer review only

For peer review only - http://bmjopen.bmj.com/site/about/guidelines.xhtml

References

1. KAISER AB, ZHANG N, DER PLUIJM WV. Global Prevalence of Type 2 Diabetes over the Next Ten Years (2018-2028). *Diabetes* 2018;67(Supplement 1):202-LB. doi: 10.2337/db18-202-LB
2. Edition IDA-t. 2017
3. Beagley J, Guariguata L, Weil C, et al. Global estimates of undiagnosed diabetes in adults. *Diabetes research and clinical practice* 2014;103(2):150-60. doi: 10.1016/j.diabres.2013.11.001
4. UK Prospective Diabetes Study (UKPDS). VIII. Study design, progress and performance. *Diabetologia* 1991;34(12):877-90. [published Online First: 1991/12/01]
5. Bommer C, Sagalova V, Heesemann E, et al. Global Economic Burden of Diabetes in Adults: Projections From 2015 to 2030. *Diabetes care* 2018;41(5):963-70. doi: 10.2337/dc17-1962 [published Online First: 2018/02/25]
6. Federation ID. IDF Diabetes Atals 9th Addition 2019;9
7. Davies MJ, D'Alessio DA, Fradkin J, et al. Management of hyperglycaemia in type 2 diabetes, 2018. A consensus report by the American Diabetes Association (ADA) and the European Association for the Study of Diabetes (EASD). *Diabetologia* 2018;61(12):2461-98. doi: 10.1007/s00125-018-4729-5 [published Online First: 2018/10/06]
8. (NICE) NICE. Diabetes in adults (Quality Standard 6) 2011 (updated 2016)
9. Federation ID. Internatioanl Standards For Educaiton Of Diabetes for Health Professional. International diabetes federation (IDF). 2015 (Last update 2017)
10. Funnell MM, Brown TL, Childs BP, et al. National standards for diabetes self-management education. *Diabetes care* 2009;32 Suppl 1:S87-94. doi: 10.2337/dc09-S087 [published Online First: 2009/01/06]
11. Stephani V, Opoku D, Beran D. Self-management of diabetes in Sub-Saharan Africa: a systematic review. *BMC public health* 2018;18(1):1148. doi: 10.1186/s12889-018-6050-0 [published Online First: 2018/10/01]
12. Federation ID. IDF Clinical Practice Recommendations for Managing Type 2 Diabetes in Primary Care *file:///Z:/Downloads/IDF-T2D-CPR-2017-interactivepdf* 2017
13. Davies MJ, Heller S, Skinner TC, et al. Effectiveness of the diabetes education and self management for ongoing and newly diagnosed (DESMOND) programme for people with newly diagnosed type 2 diabetes: cluster randomised controlled trial. *BMJ* 2008;336(7642):491-95. doi: 10.1136/bmj.39474.922025.BE
14. Gillett M, Dallosso HM, Dixon S, et al. Delivering the diabetes education and self management for ongoing and newly diagnosed (DESMOND) programme for people with newly diagnosed type 2 diabetes: cost effectiveness analysis. *BMJ* 2010;341:c4093. doi: 10.1136/bmj.c4093
15. Khunti K, Gray LJ, Skinner T, et al. Effectiveness of a diabetes education and self management programme (DESMOND) for people with newly diagnosed type 2 diabetes mellitus: three year follow-up of a cluster randomised controlled trial in primary care. *BMJ : British Medical Journal* 2012;344:e2333. doi: 10.1136/bmj.e2333
16. Harris PA, Taylor R, Thielke R, et al. Research electronic data capture (REDCap)--a metadata-driven methodology and workflow process for providing translational research informatics support. *J Biomed Inform* 2009;42(2):377-81. doi: 10.1016/j.jbi.2008.08.010 [published Online First: 2008/10/22]
17. Ritchie J SL, O'Connor W. Qualitative research practice: a guide for social science students and researchers. In: Ritchie JaLJe, ed.: London: Sage 2003:76-79.
18. Gale NK, Heath G, Cameron E, et al. Using the framework method for the analysis of qualitative data in multi-disciplinary health research. *BMC Medical Research Methodology* 2013;13(1):117. doi: 10.1186/1471-2288-13-117
19. Misra A, Gopalan H, Jayawardena R, et al. Diabetes in developing countries. *J Diabetes* 2019;11(7):522-39. doi: 10.1111/1753-0407.12913 [published Online First: 2019/03/14]

For peer review only - http://bmjopen.bmj.com/site/about/guidelines.xhtml

20. Deakin T, McShane CE, Cade JE, et al. Group based training for self-management strategies in people with type 2 diabetes mellitus. *Cochrane Database Syst Rev* 2005(2):CD003417-CD17. doi: 10.1002/14651858.CD003417.pub2
21. Chatterjee S, Davies MJ, Heller S, et al. Diabetes structured self-management education programmes: a narrative review and current innovations. *The lancet Diabetes & endocrinology* 2018;6(2):130-42. doi: 10.1016/S2213-8587(17)30239-5 [published Online First: 2017/09/29]
22. Stratton IM, Adler AI, Neil HA, et al. Association of glycaemia with macrovascular and microvascular complications of type 2 diabetes (UKPDS 35): prospective observational study. *BMJ (Clinical research ed)* 2000;321(7258):405-12. doi: 10.1136/bmj.321.7258.405
23. Wong GWK, Wright JM. Blood pressure lowering efficacy of nonselective beta-blockers for primary hypertension. *Cochrane Database Syst Rev* 2014(2):CD007452-CD52. doi: 10.1002/14651858.CD007452.pub2
24. Wong GWK, Laugerotte A, Wright JM. Blood pressure lowering efficacy of dual alpha and beta blockers for primary hypertension. *Cochrane Database Syst Rev* 2015;2015(8):CD007449-CD49. doi: 10.1002/14651858.CD007449.pub2
25. Deakin TA, Cade JE, Williams R, et al. Structured patient education: the diabetes X-PERT Programme makes a difference. *Diabet Med* 2006;23(9):944-54. doi: 10.1111/j.1464-5491.2006.01906.x
26. Steinsbekk A, Rygg LØ, Lisulo M, et al. Group based diabetes self-management education compared to routine treatment for people with type 2 diabetes mellitus. A systematic review with meta-analysis. *BMC health services research* 2012;12:213-13. doi: 10.1186/1472-6963-12-213
27. Sturt J, Dennick K, Hessler D, et al. Effective interventions for reducing diabetes distress: systematic review and meta-analysis. *International Diabetes Nursing* 2015;12(2):40-55. doi: 10.1179/2057332415Y.0000000004
28. Mandalia PK, Stone MA, Davies MJ, et al. Diabetes self-management education: acceptability of using trained lay educators. *Postgrad Med J* 2014;90(1069):638-42. doi: 10.1136/postgradmedj-2014-132865 [published Online First: 2014/09/27]

For peer review only

For peer review only - http://bmjopen.bmj.com/site/about/guidelines.xhtml

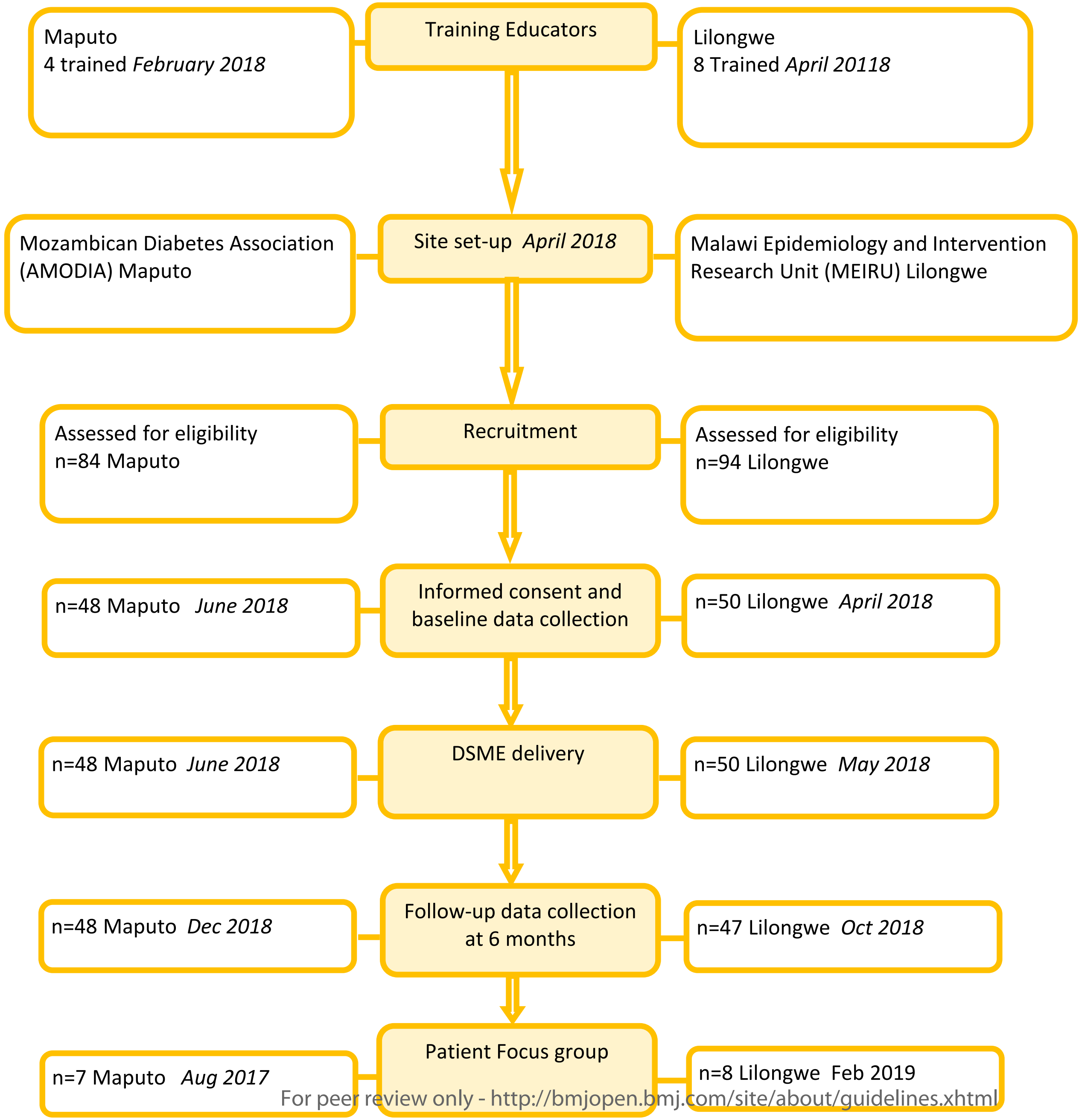
Maputo
4 trained *February 2018*
Training Educators
Lilongwe
8 Trained *April 20118*
Mozambican Diabetes Association (AMODIA) Maputo
Site set-up *April 2018*
Malawi Epidemiology and Intervention Research Unit (MEIRU) Lilongwe
Assessed for eligibility n=84 Maputo
Recruitment
Assessed for eligibility n=94 Lilongwe
n=48 Maputo *June 2018*
Informed consent and baseline data collection
n=50 Lilongwe *April 2018*
n=48 Maputo *June 2018*
DSME delivery
n=50 Lilongwe *May 2018*
n=48 Maputo *Dec 2018*
Follow-up data collection at 6 months
n=47 Lilongwe *Oct 2018*
n=7 Maputo *Aug 2017*
Patient Focus group
n=8 Lilongwe Feb 2019

EXTEND Supplementary material 1: Data collection procedures for demographic, clinical, bio-anthropometric characteristics and psychological wellbeing.

Medical History

Participants were advised to bring their health passports to both measurement sessions to assist with medical history. The following data were taken from the health passport and/or self-reported by the participant

Medical History	
Co-morbidities	Non-communicable Diseases Questionnaire (NCDQ) [1]
Diabetes Duration	Year of TD2 diagnosis for duration to be calculated (Q6042- Q6045 WHO Survey)[2]
Current medication	Taken from participant's health passports or self -report. Anti-viral medication use was to be collected on a separate CRF and stored separately to both the contacts form and main CRF (link annoymised using the unique Participant Identification number (PID)
Smoking status	Do you currently smoke any tobacco products such as cigarettes, cigars, or pipes?" and answer "Daily, yes not daily, not at all" (Q4000 WHO Survey)[2]
Alcohol	Alcohol Consumption captured using the eight questions in the STEPs survey [3]
Family history of diabetes	Provided list of family members (i.e. mother, father, siblings, paternal and maternal grandmother and grandfather) and asked whether any of them had diagnoses of T1D or T2D over their lifetime

For peer review only

Patient outcomes

Demographics	
Age	Years
Sex	Male or female
Ethnicity	*What is your [ethnic group / racial group / cultural subgroup / others] background*? Answers will come from a list of *relevant response options. (*Q1011 WHO Survey [2])
	Collect first language *"What is your mother tongue?" (*Q1000 of WHO Survey [2])
Socio-economic status	"What is the highest level of education you have completed?" (list of categories from Q1009 WHO Survey [2])
	Occupation (Q1012- Q1014 WHO Survey [2])
	Urban/rural home location will be ascertained based on address of participant

Bio- Anthropometric measurements	
Height	Measured to the nearest 0.1 cm using a portable stadiometer
Weight	Measured to the nearest 0.1 kg using a clinically approved weighing scale
Body mass index (BMI)	Calculated as weight (kg)/height (m^2)
Waist circumference	Measured with an inelastic anthropometry tape to the nearest 0.1 cm at the midpoint between the lower costal margin and iliac crest
Hip circumference	Measured at the level of the greatest protrusion of the gluteal (buttock) muscles whilst ensuring that the tape was not too tight or too loose, was lying flat on the skin, and horizontal. The participant stood erect with their weight evenly distributed on both feet and legs slightly parted, making sure not tense the gluteal (buttock) muscles

For peer review only - http://bmjopen.bmj.com/site/about/guidelines.xhtml

fasting venous blood sample	Taken by a trained Health Care Professional (HCP). Full-blood count, HbA1c, triglycerides and LDL cholesterol was measured from this sample
Blood pressure	Measured using an automated sphygmomanometer with an appropriate sized cuff while the patient was seated, and having rested quietly for 5 minutes. Three measurements were obtained for blood pressure with the average of the last two used in analysis

Health and wellbeing	
Depression	The Patient Health Questionnaire (PHQ-9) [4,5]
Diabetes related distress	PAID (Problem Areas in Diabetes Questionnaire (PAID) short form [6]
Quality of Life	The MOS short-form quality of life survey [7]
Mood	WHO-5 questionnaire commonly used to measure mental wellbeing [8]
Self-efficacy	The Self-efficacy for diabetes questionnaire is a reliable and valid 8-item scale tested in adult with diabetes [9]

Lifestyle behaviours	
Physical activity	Participants answered basic questions on physical activity from the WHO Survey (Q4030- Q4038). To supplement this, participants will also be asked to wear a GENEactiv accelerometer on their non-dominant wrist continuously (i.e. 24 hours a day) for 7 days. The devices were initialised before and downloaded after each use. Participants returned the device to the clinic at the DSME session at baseline. At follow-up they were given a stamped addressed envelope to mail it back to the research team.
Diet composition	Dietary habits were queried using questions from the Malawi STEPS survey (2009). Participants will be asked to report the number of days in the last week they have consumed at least one piece of fruit, at least one piece of veg, the number of fruit servings per day on an average day and the number of veg servings per day on an average day.

References

1. Polonsky WH, Anderson BJ, Lohrer PA, et al. Assessment of diabetes-related distress. *Diabetes care* 1995; **18**(6): 754-60.
2. https://www.who.int/healthinfo/survey/en/. 2004.
3. http://www.who.int/chp/steps/malawi/en/. 2009.
4. Kroenke K, Spitzer RL, Williams JB. The PHQ-9: validity of a brief depression severity measure. *Journal of general internal medicine* 2001; **16**(9): 606-13.
5. van Steenbergen-Weijenburg KM, de Vroege L, Ploeger RR, et al. Validation of the PHQ-9 as a screening instrument for depression in diabetes patients in specialized outpatient clinics. *BMC health services research* 2010; **10**: 235.
6. McGuire BE, Morrison TG, Hermanns N, et al. Short-form measures of diabetes-related emotional distress: the Problem Areas in Diabetes Scale (PAID)-5 and PAID-1. *Diabetologia* 2010; **53**(1): 66-9.
7. Stewart AL, Hays RD, Ware JE, Jr. The MOS short-form general health survey. Reliability and validity in a patient population. *Medical care* 1988; **26**(7): 724-35.
8. https://www.psykiatri-regionh.dk/who-5/Pages/default.aspx.
9. J S. Validity and reliability of the DMSES UK: a measure of self-efficacy for type 2 diabetes self-management. *Primary Health Care Research & Development* 2009: 1-8.

For peer review only - http://bmjopen.bmj.com/site/about/guidelines.xhtml

EXTEND Supplementary material 2: **Other medical history and family medical history**

Characteristics	Malawi n = 50	Mozambique n = 48	Overall n = 98
**Medical history**			
Wheezing/whistling chest, n (%)	2 (4·0)	4 (8·3)	6 (6·1)
Wheezing short of breath	4 (8·0)	2 (4·2)	6 (6·1)
Asthma/bronchitis, n (%)	4 (8·0)	6 (12·5)	10 (10·2)
**Family Medical History**			
Cardiovascular disease, n (%)	0 (0·0)	7 (14·6)	7 (7·1)
Stroke, n (%)	4 (8·0)	12 (25·0)	16 (16·3)
High blood pressure, n (%)	20 (40·0)	33 (68·8)	53 (54·1)
High cholesterol, n (%)	1 (2·0)	3 (6·3)	4 (4·1)
Gestational diabetes, n (%)	1 (2·0)	0 (0·0)	1 (1·0)
Type 1 Diabetes, n (%)	7 (14·0)	0 (0·0)	7 (7·1)
Type 2 Diabetes, n (%)	17 (34·0)	28 (58·3)	45 (45·9)
Depression, n %)	0 (0·0)	4 (8·3)	4 (4·1)
Sleep Disorder, n (%)	0 (0·0)	6 (12·5)	6 (6·1)

Please note: A diagnosis of depression was not collected from participants· % percent

For peer review only - http://bmjopen.bmj.com/site/about/guidelines.xhtml

EXTEND Supplementary material 3: Type of medication

	Baseline			Follow-up		
**Type of Medication**	**Malawi**	**Mozambique**	**Overall**	**Malawi**	**Mozambique**	**Overall**
	n = 50	n = 48	n = 98	n = 47	n = 47	n = 94
Diet and life style only	None	None	None	None	None	None
Mono or combination therapy, n (%)						
Mono therapy						
Metformin	4 (8·0)	25 (52·1)	29 (29·6)	6 (12·8)	12 (25·5)	18 (19·1)
Sulfonylurea	1 (2·0)	5 (10·4)	6 (6·1)	0 (0·0)	3 (6·4)	3 (3·2)
Insulin	0 (0·0)	5 (10·4)	5 (5·1)	0 (0·0)	6 (12·8)	6 (6·4)
Dual therapy						
Metformin + Sulfonylurea	45 (90·0)	5 (10·4)	50 (51·0)	41 (87·2)	12 (25·5)	53 (56·4)
Metformin + Insulin	0 (0·0)	8 (16·7)	8 (8·2)	0 (0·0)	14 (29·8)	14 (14·9)
Anti-hypertension medication, n (%)	36 (72·0)	29 (60·4)	65 (66·3)	31 (62·0)	32 (66·7)	63 (67·0)
Diuretics	26 (52·0)	22 (45·8)	48 (49·0)	16 (34·0)	19 (40·4)	35 (37·2)
Calcium channel blocker	22 (44·0)	19 (39·6)	41 (41·8)	25 (53·2)	20 (42·6)	45 (47·9)
ACE-Inhibitor	8 (16·0)	16 (33·3)	24 (24·5)	3 (6·4)	15 (31·9)	18 (19·1)
Beta blocker	4 (8·0)	8 (16·7)	12 (12·2)	3 (6·4)	7 (14·9)	10 (10·6)
Centrally acting anti-hypertensive	0 (0·0)	1 (3·5)	1 (1·0)	1 (2·1)	2 (4·3)	3 (3·2)
Aspirin	1 (2·0)	0 (0·0)	1 (1·0)	1 (2·1)	0 (0·0)	1 (1·1)
Lipid lowering medication, n (%)						
Statins (Simvastatin)	0 (0·0)	13 (27·1)	13 (13·3)	0 (0·0)	12 (25·0)	12 (12·8)

Please note: Medication adherence was not collected· Data provided count (%)

For peer review only

EXTending availability of self-management structured EducatioN programmes for people with type 2 Diabetes in low-to-middle income countries (EXTEND) – A feasibility study in Mozambique and Malawi

Journal:	*BMJ Open*
Manuscript ID	bmjopen-2020-047425.R1
Article Type:	Original research
Date Submitted by the Author:	02-Jun-2021

Complete List of Authors:	Brady, Emer M.; University Hospitals of Leicester NHS Trust, Leicester Diabetes Centre<br>Bamuya, Catherine; Malawi Epidemiology and Intervention Research Unit<br>Beran, David; Division of Tropical and Humanitarian Medicine<br>Correia, Jorge ; University Hospitals Geneva, Unit of Patient Education, Division of Endocrinology<br>Crampin, Amelia; London School of Hygiene and Tropical Medicine<br>Damasceno, Albertino; Universidade Eduardo Mondlane<br>Davies, Melanie; University of Leicester, Diabetes Research Centre<br>Hadjiconstantinou , M ; University of Leicester, Diabetes Research Centre<br>Harrington, Deirdre; University of Leicester, Diabetes Research Centre<br>Khunti, Kamlesh; University of Leicester, Diabetes Research Centre<br>Levitt, Naomi; University of Cape Town<br>Magaia, Ana; Eduardo Mondlane University, Faculty of Medicine<br>Mistry, Jayna; University Hospitals of Leicester NHS Trust, Leicester Diabetes Centre<br>Namadingo, Hazel; Malawi Epidemiology and Intervention Research<br>Rodgers, Anne; University Hospitals of Leicester NHS Trust, Leicester Diabetes Department<br>Schreder, Sally; University Hospitals of Leicester NHS Trust, Leicester Diabetes Centre<br>Simango , Leopoldo ; Mozambican Diabetes Association (AMODIA)<br>Stribling , Bernie; University Hospitals of Leicester NHS Trust, Leicester Diabetes Centre<br>Taylor, Cheryl; University Hospitals of Leicester NHS Trust, Leicester Diabetes Centre<br>Waheed, Ghazala; University of Leicester, Health Sciences

**Primary Subject Heading**:	Diabetes and endocrinology
Secondary Subject Heading:	Patient-centred medicine
Keywords:	DIABETES & ENDOCRINOLOGY, MEDICAL EDUCATION & TRAINING, EDUCATION & TRAINING (see Medical Education & Training), PREVENTIVE MEDICINE

I, the Submitting Author has the right to grant and does grant on behalf of all authors of the Work (as defined in the below author licence), an exclusive licence and/or a non-exclusive licence for contributions from authors who are: i) UK Crown employees; ii) where BMJ has agreed a CC-BY licence shall apply, and/or iii) in accordance with the terms applicable for US Federal Government officers or employees acting as part of their official duties; on a worldwide, perpetual, irrevocable, royalty-free basis to BMJ Publishing Group Ltd ("BMJ") its licensees and where the relevant Journal is co-owned by BMJ to the co-owners of the Journal, to publish the Work in this journal and any other BMJ products and to exploit all rights, as set out in our licence.

The Submitting Author accepts and understands that any supply made under these terms is made by BMJ to the Submitting Author unless you are acting as an employee on behalf of your employer or a postgraduate student of an affiliated institution which is paying any applicable article publishing charge ("APC") for Open Access articles. Where the Submitting Author wishes to make the Work available on an Open Access basis (and intends to pay the relevant APC), the terms of reuse of such Open Access shall be governed by a Creative Commons licence – details of these licences and which Creative Commons licence will apply to this Work are set out in our licence referred to above.

Other than as permitted in any relevant BMJ Author's Self Archiving Policies, I confirm this Work has not been accepted for publication elsewhere, is not being considered for publication elsewhere and does not duplicate material already published. I confirm all authors consent to publication of this Work and authorise the granting of this licence.

For peer review only

EXTending availability of self-management structured EducatioN programmes for people with type 2 Diabetes in low-to-middle income countries (EXTEND) – A feasibility study in Mozambique and Malawi

Emer M Brady PhD[1]; The EXTEND Collaborative

Catherine Bamuya MSc[2], David Beran PhD[3], Jorge C Correia MD[4], Amelia C Crampin [2], Albertino Damasceno PhD[5], Melanie J Davies MD[1,6], Michelle Hadjiconstantinou PhD [1,6], Deirdre M. Harrington PhD [6,9], Kamlesh Khunti MD[1,6],Naomi Levitt [7], Ana Magaia MD[5], Jayna Mistry BA[1], Hazel Namadingo MSc[2], Anne Rodgers BSc[1], Sally Schreder MSc [1], Leopoldo Simango MPH [8], Bernie Stribling MSc [1],Cheryl Taylor MSc[1], Ghazala Waheed MSc[1,6].

Affiliations:

[1] Leicester Diabetes Centre, University Hospitals of Leicester NHS Trust UK

[2] Malawi Epidemiology and Intervention Research Unit, Malawi

[3] Division of Tropical and Humanitarian Medicine, Faculty of Medicine, University of Geneva and Geneva University Hospitals, Switzerland

[4] Unit of Patient Education, Division of Endocrinology, Diabetology, Nutrition and Patient Education, WHO Collaborating Center, Department of Medicine, University of Geneva and Geneva University Hospitals, Geneva, Switzerland

[5] Faculty of Medicine, Eduardo Mondlane University, Mozambique

[6] Diabetes Research Centre, College of Life Sciences, University of Leicester, UK

[7] University of Cape Town, Cape Town, South Africa

[8] Mozambican Diabetes Association, Maputo, Mozambique

[9] School of Psychological Sciences and Health, University of Strathclyde, UK

Corresponding author

Dr Emer M Brady
Cardiovascular Sciences
College of Life Sciences
University of Leicester
UK
emb29@le.ac.uk

Word count (minus abstract): 3,485

For peer review only - http://bmjopen.bmj.com/site/about/guidelines.xhtml

Abstract (244)

Background

Globally, there are estimated 425 million people with Type 2 Diabetes (T2D) with 80% from Low-Middle Income Countries (LMIC). Diabetes Self-Management Education (DSME) Programmes are a vital and core component of the treatment pathway for T2D. Despite LMIC being disproportionally affected by T2D, there are no DSME available that meet international diabetes federation criterion.

Methods

The aims were to test the feasibility of delivering a proven effective and cost-effective approach used in a UK population in two urban settings in Malawi and Mozambique by; 1) developing a culturally, contextually and linguistically adapted DSME, the EXTEND programme; 2) using a mixed-method approach to evaluate the delivery of training and the EXTEND programme to patients with T2D.

Results

Twelve healthcare professionals were trained. Ninety-eight participants received the DSME. Retention was high (100% in Mozambique and 94% in Malawi). At 6 months HbA1c (-0.9%), cholesterol (-0.3mmol/L), blood pressure (-5.9mmHg systolic and -6.1mmHg diastolic) improved in addition to indicators of wellbeing (problem areas in diabetes and self-efficacy in diabetes).

Conclusion

It is feasible to deliver and evaluate the effectiveness of a culturally, contextually and linguistically adapted EXTEND programme in two LMIC. The DSME was acceptable with positive biomedical and psychological outcomes but requires formal testing with cost-effectiveness. Challenges exist in scaling up such an approach in health systems that do not have resources to address the challenge of diabetes.

For peer review only - http://bmjopen.bmj.com/site/about/guidelines.xhtml

Strengths and Limitations of this study

1) This was not a randomised controlled trial but a feasibility study that included patient and patient related outcome measures
2) No control group was included in this feasibility study as it was considered unethical at this stage to deny people with Type 2 diabetes (T2D) the diabetes self-management education (DSME) programme
3) Standard operating procedures were created and utilised throughout the study for all data capture
4) Data were double entered (and discrepancies corrected with source data) from paper case report forms into a secure web-based database specifically designed for the study
5) Patients with T2D and healthcare professionals involved in delivering their care had direct involvement in the cultural and linguistic adaptation of the UK based DSME at each location

For peer review only

For peer review only - http://bmjopen.bmj.com/site/about/guidelines.xhtml

Introduction

The global estimate of prevalent cases of type 2 Diabetes (T2D) is approximately 500 million [1], and accounts for 10.7% of all-cause mortality in people aged between 20-79 years old[2]. There are a further 212 million people thought to be undiagnosed [2] with an overall disproportionate number of cases seen in low-middle income-countries (LMIC) [3].

T2D is a progressive chronic condition, when sub-optimally managed, can lead to the development of both microvascular and macrovascular complications, including for example retinopathy, nephropathy, and neuropathy, heart disease, peripheral vascular disease, resulting in end organ damage in approximately one third to one half of people with T2D [4]. This is concerning particularly in those health systems, such as in LMICs,that are fragmented, overstretched and under resourced, with intermittent drug supplies and rudimentary clinical training on T2D.

The vast economic burden of T2D includes both direct costs from medical care and indirect costs via loss of productivity or earnings, summing to some estimated $1.3 trillion [5]. It is estimated that the healthcare cost for a person with diabetes is two-fold higher than without diabetes. This global public health issue is impacting the world's poorest countries; indeed 80% of all cases of T2D come from LMIC such as those within Sub-Saharan Africa (SSA). The current epidemiological transition occurring in SSA, due to rapid urbanisation and nutritional shift, has seen an increase in the burden of T2D[2]. It is predicted that by 2045, 47.1 million people in SSA will have T2D[6]. The additional and increasing strain of T2D placed on their already stretched health systems highlights the need to find cost-effective approaches to reducing the disease burden.

Leading international health organisations promote self-management structured education as the cornerstone of diabetes care, and recommend Diabetes Self-Management Education (DSME) programmes as a core component of the treatment pathway for diabetes[7-9]. The overall objectives of

For peer review only - http://bmjopen.bmj.com/site/about/guidelines.xhtml

DSME are to support informed decision-making, self-care behaviours, problem-solving and active collaboration with the health care team and to improve clinical outcomes, health status, and quality of life [10]. DSME offer a potential financially viable treatment option for healthcare settings within both high-income countries (HIC) and LMIC.

A recent systematic review highlighted that despite there being a number of studies looking at self-management behaviours in SSA, self-management itself is insufficient in these countries. In particular patients do not engage, or are aware in some cases, of risk reducing behaviours such as physical activity, reducing salt-intake and good foot care [11]. Furthermore, the DSME described in these studies do not meet the standards set-out by the National Institute of Clinical Excellence (NICE) in the UK, that is, that they include certain components [9] for example;

- an evidence-base
- suits the needs of the person
- has specific learning objectives
- that supports the person in developing attitudes, beliefs, knowledge and skills to self-manage diabetes
- have a structured curriculum that is theory-driven, evidence-based and resource-effective with supporting materials, and is written down
- Delivered by trained educators
- is quality assured

These principles are supported by the American Diabetes Association, European Association for the Study of Diabetes [7] and the International Diabetes Federation [8]. To our knowledge there are no DSME programmes with proven effectiveness and cost-effectiveness in SSA that meet these criteria.

For peer review only - http://bmjopen.bmj.com/site/about/guidelines.xhtml

The EXTEND programme was a cultural and contextual adaptation of a UK DSME that meets international criteria for DSME and has previously been shown to be effective and cost-effective in people with T2D. The aim of this study was to test the feasibility of the EXTEND programme including; working with local teams to deliver training, recruiting patients, delivering the programme and collecting biomedical and psychological research outcomes in two SSA urban settings in Malawi (Lilongwe) and Mozambique (Maputo).

Methods

This was a single group feasibility study with mixed-methods evaluation. All participants received the intervention. This study was funded by the Global Challenges Research Fund NCDs Foundation Awards 2016 Developmental Pathway Funding (Medical Research Council (MR/P02548X/1)). All participants were offered the intervention. Data were collected after informed consent was obtained and before the intervention (baseline) and at 6 months. The detailed CONSORT diagram is provided in supplemental material 1. Briefly in Lilongwe baseline data were collected in April 2018, DSME delivered in May 2018 and follow-up data in October 2018. In Maputo baseline data and DSME delivery took place in June 2018 and follow-up in December 2018. The qualitative study was conducted in Lilongwe February 2019 and in August 2019 in Maputo.

Ethics Approval

Ethical approval was granted by: the Scientific Commission of the Faculty of Medicine and the Mozambique National Research Ethics Committee CNBS – Comité National de Bioética para Saúde, (CIBS FM&HCM/80/2017); the College of Medicine Research Ethics Committee, Malawi (P.10/17/2301); and the University of Leicester College of Life Sciences research ethics committee (ref:145734, 04/04/2018).

For peer review only - http://bmjopen.bmj.com/site/about/guidelines.xhtml
For peer review only

Participants

Patients were recruited from the private diabetes out-patients clinic provided by the Mozambican Diabetes Association (AMODIA), in Maputo. In Malawi patients were identified from the government funded health centre in Area 25 in Lilongwe. In both settings the patients' paper health records were examined for eligibility. Eligibility were a diagnosis of T2D and 18 years old or older. Exclusion criteria were: severe and enduring mental health problems; not primarily responsible for their own care, could not provide informed consent; not able to participate in activities in a group setting; or currently participating in another intervention study. Those identified as eligible were either approached at their next appointment or telephoned by one of the study researchers. The study was explained and if they were interested they were invited to attend the baseline assessment visit. All participants had at least 24 hours to consider participation and could withdraw at any time without their usual care being affected.

Sample size

No formal sample size was calculated because this is a feasibility study. A sample size of 50 participants in each site was selected based on a balance between pragmatism and having a large enough sample to produce reasonable parameter estimates to power a future formal evaluation of the EXTEND programme and experience of the logistics of its delivery.

The intervention

The DSME programme, named "EXTEND", aimed to extend the availability of self-management structured education programme for people with T2D in LMIC. EXTEND is an interactive group based programme culturally and contextually adapted from a programme first developed and tested in the UK [12-14] and meets international guidelines for DSME. It was delivered in two 3-hour sessions by two

For peer review only - http://bmjopen.bmj.com/site/about/guidelines.xhtml

trained educators to people with T2D in Portuguese in Maputo and Chicheŵa in Lilongwe. The DSME was delivered within three weeks of the baseline data collection visit.

The programme has a written curriculum and educators were trained to elicit the learning of the participants by adopting a non-didactic approach to the group learning. A large part of the curriculum is focused on lifestyle factors, such as food choices, physical activity, and cardiovascular risk factors. The UK DSME (DESMOND) and thus the EXTEND programme aimed to activate the participants to explore their own personal risk factors and from this generate achievable goal(s) with an action plan whilst considering barriers and enablers. The whole programme is underpinned by several learning and behaviour change theories including; the dual process theory, self-efficacy, the social learning theory and Leventhal's common sense theory as described by Skinner et al[15].

Patient and Public Involvement

Patient and public involvement was of critical importance during the adaptation of the UK based DSME programme. The EXTEND programme was co-produced by the EXTNED investigators and patients, educators, nurses and patients spouses/children. The UK DSME with supporting resources were taken to Mozambique and Malawi separately by the national educators. At site the local research teams had invited patients and those who would eventually be trained to deliver the education to be part of the PPI group and attend a two-day session where all content were shared and scrutinised by the local PPI group. Sections of the DSME that were not relevant to the local population were removed or amended. Local foods and cooking practices were included as directed by the PPI groups and other topics of importance for example erectile dysfunction and natural remedies for diabetes. The adaptations were made back in the UK in collaboration with the local researchers. The DSME was then translated which prompted further amendments given differences on vernacular. The UK national educators then returned the each locality and delivered EXTEND to two new groups of patients and their spouse/child and received further alterations. During the delivery of EXTEND in the study further

For peer review only - http://bmjopen.bmj.com/site/about/guidelines.xhtml

suggestions made during the study were collected and feedback and incorporated into the final version. Please see supplementary material 2 for key adaptations.

Educator training

Educators who delivered EXTEND were trained by accredited national educator trainers from the UK who conducted four-day training sessions in each of the settings; a total of eight educators were successfully trained in Lilongwe and four in Maputo (supplementary material 3). The trainers' set-up a WhatsApp group with each group of educators to provide on-going support remotely as requested by the educators. In Malawi the people trained consisted of; 3 nurses and 5 lay people with T2D. The research associate at location selected 4 of the most competent educators to take part in the feasibility study which included 2 nurses and 2 lay people with T2D. The remaining 4 went back into their communities to spread the messages. In Mozambique the people trained included 3 nurses and a medical student all 4 took part in the feasibility study.

Participants and educators were provided refreshments and refunded travel expenses at both baseline, education and follow-up visits.

Feasibility related outcomes

Feasibility related outcomes were collected via a recruitment log completed by the onsite recruitment team:

1. Number of eligible patients referred who accepted the invitation and number who refused
2. Number of eligible patients referred who accepted the feasibility study invitation and attended DSME and research study visits (baseline only, baseline and follow-up)
3. Data collected at each visit
4. Baseline characteristics of the sample who were enrolled in the study
5. Retention rate
6. Change values for each of the potential outcome measures

For peer review only - http://bmjopen.bmj.com/site/about/guidelines.xhtml

Participant outcome data

Pre and post data were collected from consenting participants at baseline and 6 months. Data were recorded in a paper-based case report form (CRF). Standard operating procedures (SOPs) were developed, agreed and followed at both sites. Data were double entered into a REDCap [16] database, which uses a 'My Structured Query Language' (MySQL) database (an open-source relational database management system (RDBMS)) via a secure web interface, with data checks used during data entry to ensure quality. All supporting systems were hosted and housed within the secure networked environment provided by the University of Leicester, UK.

Data collected are provided in *supplementary material 4* but in summary include: demographics, medical history, anthropometric measurements, cardio-metabolic related outcomes, psychological outcomes pertaining to health and wellbeing and lifestyle behaviours.

Qualitative study

The qualitative study was conducted in both settings with the purpose of exploring the views and experiences of those directly involved with the EXTEND programme (i.e. people with T2D, trainers, educators). In addition, the views of clinicians and stakeholders who are regularly in contact with people with T2D were explored on the potential for future implementation. Findings from this qualitative study are presented in detail elsewhere. Briefly, focus group discussions were conducted in the Faculty of Medicine premises (Maputo), and in Area 25 health centre (Lilongwe) (August 2018 to April 2019) with discussions lasted approximately 90 minutes. The focus groups were carried out by our research team (MH, CB, JCC) and audio recorded. MH, who has extensive experience in qualitative research, led data collection and analysis. Where required, research members also acted as translators (JCC).

Here, an overview of the participants' views on and experience with the EXTEND programme is provided.

For peer review only - http://bmjopen.bmj.com/site/about/guidelines.xhtml

Analysis

Quantitative data analysis

Descriptive statistics were produced for participant characteristics at baseline using mean (SD) for normally distributed variables, median (IQR) for non-normally distributed variables, count and percentage (%) for categorical variables. Each of the outcome measures has been summarised using appropriate descriptive statistics at baseline and at 6 months. A paired t test for normally distributed continuous variables (or Mcnemar's test for categorical variables) was used to compare baseline and post intervention means (or proportions) separately in each country. Non-parametric Wilcoxon ranksum test was used to compare baseline and follow-up medians when the data were not normally distributed.

Qualitative data analysis

Taking an inductive thematic approach, data was analysed by two researchers (MH and CB) based on the Framework method [17] and applying principles of the constant comparative techniques [18]. Data was organised with the use of the NVivo qualitative data indexing software. An initial coding framework was generated, and further refined through additional coding against transcripts. Data were subsequently summarised and exported into matrices to enable comparison of themes systematically. To ensure credibility, we used investigator triangulation[19], whereby the two researchers (MH and CB) coded and analysed the data for both localities.

Results

Recruitment and retention

A total of 122 were invited to participate across both sites with a total of 12 declining to participate. Reasons for declining included a lack of time, deficiencies for authorization of the work place, unwillingness to attend on Saturday due to family ceremonies, difficulties in communicating in

For peer review only - http://bmjopen.bmj.com/site/about/guidelines.xhtml

Portuguese. A total of 98 participants, 50 in Malawi and 48 in Mozambique were recruited to the feasibility study. Overall the mean age was 55.2 years and 62% were female. The retention rate was high (Supplementary material 1). All outcome data were successfully collected in both sites for both study time-points with the exception of the objective measure of physical activity, which was collected in Malawi at baseline only. In pairs the educators delivered EXTEND to all participants.

In Lilongwe all data were collected and the first education session delivered at Malawi Epidemiology and Intervention Research Unit (MEIRU) premises where participants were asked to travel for a distance of about 20kms. The participants requested the second education session be delivered at the Area 25 health clinic due to reduced travel time and expense and increased accessibility. Participants preferred all appointments in the afternoon. The data were collected by the research associated assigned to the study with support from a nurse. In Maputo, all data were collected and the education delivered at the AMODIA out patients' clinic situated on the grounds of the Hospital Central de Maputo in the city centre. The data were collected by the two research associates assigned to the study. Data from both sites were recorded in a paper-based case report form (CRF). Data were double entered into a REDCap database, which uses a 'My Structured Query Language' (MySQL) database (an open-source relational database management system (RDBMS)) via a secure web interface, with data checks used during data entry to ensure data quality. All supporting systems were hosted and housed within the secure networked environment provided by the University Of Leicester, UK.

Baseline characteristics (Table 1)

Twenty-two per cent of participants were within the accepted "normal" range for BMI, 35% were overweight and 43% as obese. Overall, between 60-78% had hypertension with a low prevalence of hypercholesterolemia in Malawi compared to 41% of the Mozambican cohort. A family history of T2D and/or hypertension was common (supplementary material 5). HbA1c was similar between the two cohorts and indicative of poor glycaemic control. Fasting glucose was higher in Mozambique but in

For peer review only - http://bmjopen.bmj.com/site/about/guidelines.xhtml

both settings was > 7.0mmol/L, again indicating sub-optimal control. Despite total cholesterol levels being within the 'healthy' range HDLc was low "normal" and LDLc was high "normal" which are considered to be associated with increased risk of heart disease. Current medication is provided in supplementary material 6; no participants were managing their T2D with diet and lifestyle only. The majority were on dual therapy of metformin and sulphonylurea and none on insulin therapy at the time of the study in Malawi. In Mozambique the majority were using monotherapy (>70%). Over 60% in both cohorts were taking anti-hypertensive medication and a quarter of those in Mozambique were on lipid-lowering medication. No participants in Malawi reported being prescribed lipid lowering medication. Data were not collected on medicine adherence. There was a shift from monotherapy (metformin) to dual therapy from baseline to follow-up; metformin plus sulphonylurea increased by 5% and metformin plus insulin by 7%. There was a small reduction in those receiving a diuretic at six months.

For peer review only

For peer review only - http://bmjopen.bmj.com/site/about/guidelines.xhtml

Table 1| Baseline characteristics

	Malawi n = 50	Mozambique n = 48	Overall n = 98
**Demographics**			
Age	56.2 (11.6)	54.2 (7.8)	55.2 (9.9)
Gender (female)	30 (60.0)	31 (64.6)	61 (62.2)
Duration T2DM (years)	6.8 (5.5)	8.81(5.9)	7.79(5.8)
**Medical History (n, %)**			
Hypertension	39 (78.0)	29 (60.4)	68 (69.4)
High cholesterol	2 (4.0)	20 (41.7)	22 (22.5)
Last time cholesterol checked (months)*	0 (0 – 9)	1 (1 – 2)	1 (0 – 3)
Stroke	5 (10.0)	1 (2.1)	6 (6.1)
Heart disease	0 (0.0)	1 (2.1)	1 (1.0)
Tuberculosis	4 (8.0)	4 (8.3)	8 (8.2)
**Biomedical characteristics**			
HbA1c (%)*	9.7 [7.9 –14.7]	9.6 [7.6 –14.7]	9.6[7.7– 14.7]
HbA1c (mmol/mol)*	102.6 (48.4)	95.1 (46.5)	98.9 (47.4)
Fasting glucose (mmol/L)*	7.5 [5.5 –10.3]	9.0 [6.5 –13.2]	8.1[6.2– 12.0]
Total cholesterol (mmol/L) **	5.1 (1.3)	4.7 (0.9)	4.9 (1.2)
HDL (mmol/L)**	1.3 (0.3)	1.2 (0.3)	1.2 (0.3)
LDL (mmol/L) **	3.4 (1.0)	2.8 (0.9)	3.1 (1.0)
Triglycerides (mmol/L)*	1.5 [1.0 – 1.9]	1.1 [0.8 – 1.6]	1.3[0.9-1.8]
Systolic BP (mmHg) **	136.8 (20.6)	142.8 (22.6)	139.7 (21.7)
Diastolic BP (mmHg)**	86.8 (8.9)	89.6 (10.8)	88.2 (9.9)
Weight (Kg) **	71.2 (13.9)	82.2 (17.8)	76.5 (16.7)
BMI (Kg/m^2) **	27.4 (5.3)	29.5 (6.4)	28.5 (6.0)
**BMI categories**			
Normal (20-24.9 Kg/m^2)**	11 (23.4)	10 (21.3)	21 (22.3)
Overweight (25-29.9 Kg/m^2)**	19 (40.4)	14 (29.8)	33 (35.1)
Obese (≥ 30 Kg/m^2)**	17 (36.2)	23 (48.9)	40 (42.6)
Waist circumference (cm)**	91.8 (12.6)	95.6 (15.5)	93.6 (14.2)

* Median [Interquartile range [IQR]] for non-normally distributed variables, ** Mean (SD) for normally distributed variables.
HbA1c: Glycated haemoglobin; HDL: High Density Lipoprotein; LDL: Low Density Lipoprotein; BMI: Body Mass Index

For peer review only

For peer review only - http://bmjopen.bmj.com/site/about/guidelines.xhtml

Changes in biomedical and psychological outcomes from baseline (Table 2 and 3, respectively)

Although, this feasibility study was not powered to detect statistically significant differences in biomedical outcomes, overall the reductions in HbA1c (-0.9% (95%CI: -1.4 to-0.1)), total cholesterol (-0.3mmol/L (95%CI: -0.4 to -0.1)), LDL cholesterol (-0.2mmol/L (95%CI: -0.3 to -0.0)), triglycerides (-0.2mmol/L(95%CI: -0.3 to -0.1)), diastolic (-6.1mmHg (95%CI: -8.2 to -4.0)) and systolic (-5.9mmHg, (95%CI: -9.6 to -2.1)) blood pressure are clinically important and do reach statistical significance. Heart rate, weight and BMI increased negligibly in both cohorts. A clinically relevant reduction in HbA1c, glucose and diastolic blood pressure are observed in participants in Malawi with modest reductions also observed for total cholesterol, LDL cholesterol and weight at six months follow-up. The same pattern is seen in participants from Mozambique in addition to a significant reduction in systolic blood pressure.

Overall the prevalence of 'non-minimal presence of depressive symptoms' (as measured by PHQ-9) at baseline was high at 52% and increased at six months. There were no reported cases of severe depressive symptoms in either cohort (score 20-27). Malawi and Mozambique had similar levels of mild and moderate depression at baseline (Malawi 32% and 10% respectively, Mozambique 39.6% and 10.4%, respectively) with a lower prevalence for both categories at 6 months. The Problem Areas in Diabetes (PAID) showed a significant improvement with an overall reduction of approximately 7 points at six months i.e. a lower score is indicative of fewer problems associated with diabetes. A comparable reduction was observed between the two time-points in each setting (Malawi 5 points and Mozambique approximately 9 points). Overall wellbeing (WHO (Five)) was high in both settings and remained essentially unchanged in Malawi but there was a reduction in the proportion of those likely to have depression (score <25) in Mozambique at 6 months. Overall self-efficacy for diabetes improved at six months reaching statistical significance. Results from the 20-Item Short Form Health Survey (SF-20) indicated a high and maintained level of physical, role and social functioning. In addition

For peer review only - http://bmjopen.bmj.com/site/about/guidelines.xhtml

a reduction in pain and statistically significant improvement in health perceptions at six months was observed.

For peer review only

For peer review only - http://bmjopen.bmj.com/site/about/guidelines.xhtml

Table 2 | Mean change in biomedical characteristics from baseline

	Malawi				Mozambique				Overall			
	**Baseline**	**Follow-up**	**Mean change (95% CI) n = 47**	**P - value**	**Baseline**	**Follow-up**	**Mean change (95%CI) n = 48**	**P- value**	**Baseline**	**Follow-up**	**Mean change (95%CI) n = 95**	P-value
**Characteristic**												
HbA1c (%) **	9.7 [7.9-14.7]	9.8 [7.6-13.3]	-0.8 (-1.3 to 0.1)	0.058	9.6 [7.6-14.7]	8.4 [7.2-10.7]	-1.1 (-2.5 to 0.1)	0.144	9.6 [7.7-14.7]	8.8 [7.4-12.1]	-0.9 (-1.4 to-0.1)	0.001
HbA1c (mmol/mol)	102.6 (48.4)	89.5 (39.5)	9.7 (32.2)		95.1 (46.5)	77.3 (31.1)	17.8 (39.3)		98.9 (47.4)	83.4 (35.8)	13.8 (35.9)	
Glucose (mmol/L)**	7.5 [5.5-10.3]	7.6 [5.6-10.7]	-0.4 (-0.9 to 0.4)	0.589	9.0 [6.5-13.2]	7.7 [5.8-12.4]	-0.9 (-1.9 to 0.4)	0.372	8.1 [6.2-12.0]	7.6 [5.7-12.1]	-0.4 (-1.1 to 0.03)	0.313
Total cholesterol (mmol/L)	5.1 (1.3)	4.9 (1.2)	-0.2 (-0.4 to -0.0)	0.046	4.7 (0.9)	4.4(1.0)	-0.3 (-0.6 to -0.1)	0.016	4.9 (1.2)	4.6 (1.1)	-0.3 (-0.4 to -0.1)	0.002
HDL (mmol/L)	1.3 (0.3)	1.3 (0.3)	-0.1 (-0.1 to 0.0)	0.069	1.2 (0.3)	1.3 (0.4)	0.1 (-0.0 to 0.1)	0.087	1.2 (0.3)	1.3 (0.3)	0.0 (-0.1 to 0.1)	0.997
LDL (mmol/L)	3.4 (1.0)	3.3 (0.9)	-0.2 (-0.3 to-0.0)	0.017	2.8 (0.9)	2.6 (0.8)	-0.2 (-0.4 to 0.1)	0.147	3.1 (1.0)	2.9 (0.9)	-0.2 (-0.3 to -0.0)	0.013
Triglycerides (mmol/L)**	1.5 [1 – 1.9]	1.3 [0.9 -2.0]	-0.2 (-0.4 to -0.1)	0.008	1.1 [0.8 - 1.6]	1.0 [0.8 -1.3]	-0.1(-0.3 to 0.1)	0.122	1.3 [0.9 – 1.8]	1.2 [0.8-1.7]	-0.2 (-0.3 to -0.1)	0.002
Systolic BP (mmHg)	136.8 (20.6)	138.5 (19.3)	0.7 (-3.4 to 4.8)	0.736	142.8 (22.6)	130.4 (18.9)	-12.3 (-18.2to -6.5)	0.000	139.7 (21.7)	134.4 (19.4)	-5.9 (-9.6 to -2.1)	0.003
Diastolic BP (mmHg)	86.8 (8.9)	80.8 (8.9)	-6.1 (-8.9 to -3.3)	0.000	89.6 (10.8)	83.5 (9.2)	-6.1 (-9.4 to -2.8)	0.001	88.2 (9.9)	82.2 (9.1)	-6.1 (-8.2 to -4.0)	<0.001
Heart rate (bpm)	79.7 (14.5)	80.9 (12.8)	1.3 (-2.1 to 4.7)	0.437	74.3 (12.4)	76.3 (10.6)	1.9 (-1.5 to 5.4)	0.269	77.1 (13.7)	78.6 (11.9)	1.6 (-0.8 to 4.0)	0.178
Weight (Kg)	71.1 (13.9)	71.0 (12.4)	0.1 (-0.9 to 1.1)	0.809	82.2 (17.8)	82.0 (17.2)	-0.2 (-1.3 to 1.0)	0.781	76.5 (16.7)	76.5 (15.9)	-0.0 (-0.7 to 0.7)	0.595
Waist (cm)	91.8 (12.6)	91.0 (11.7)	-0.9 (-2.8 to 0.9)	0.323	95.6 (15.5)	98.0 (14.4)	2.4 (-0.2 to 4.9)	0.067	93.6 (14.2)	94.5 (13.5)	0.8 (-0.8 to 2.4)	0.345
BMI (Kg/m^2)	27.4 (5.4)	27.5 (4.9)	0.03 (-0.3 to 0.4)	0.880	29.6 (6.4)	29.5 (6.2)	-0.1(-0.5 to 0.3)	0.747	28.5 (6.0)	28.5 (5.7)	-0.0(-0.3 to 0.3)	0.892

All values are mean (Standard deviation (SD) for normally distributed variables), unless otherwise stated **Median change [range] for non-normally distributed variables

HbA1c: Glycated haemoglobin; HDL: High Density Lipoprotein; LDL: Low Density Lipoprotein; BMI: Body Mass Index

review only

For peer review only - http://bmjopen.bmj.com/site/about/guidelines.xhtml

Table 3 | Scores for measures from questionnaire at baseline and follow-up

Questionnaire Score	Malawi Baseline N = 50	Malawi Follow-up N = 47	P-value*	Mozambique Baseline N = 48	Mozambique Follow-up N = 48	P-value*	Overall Baseline N = 98	Overall Follow-up N = 95	P-value*
Health and Wellbeing related outcomes (PHQ-9) [a]									
Depressive symptoms severity									
None-minimal (0-4), n (%)	27 (54.0)	33 (70.2)	0.134	24 (50.0)	29 (60.4)	0.297	51 (52.0)	62 (65.3)	0.078
Mild (5-9), n (%)	16 (32.0)	11 (23.4)	0.317	19 (39.6)	15 (31.3)	0.414	35 (35.7)	26 (27.4)	0.206
Moderate (10-14), n (%)	5 (10.0)	3 (6.4)	1.000	5 (10.4)	3 (6.3)	0.480	10 (10.2)	6 (6.3)	0.527
Moderately severe (15-19), n (%)	2 (4.0)	0 (0.0)	0.157	0 (0.0)	1 (2.1)	0.317	2 (2.0)	1 (1.1)	0.564
Severe (20-27), n (%)	0 (0.0)	0 (0.0)	-	0 (0.0)	0 (0.0)	-	0 (0.0)	0 (0.0)	-
Proportion with score > 10, n (%)	6 (12.0)	1 (2.1)	0.059	2 (4.2)	3 (6.3)	0.564	8 (8.2)	4 (4.2)	0.206
Proportion with score ≥ 10, n (%)	7 (14.0)	3 (6.0)	0.103	5 (10.4)	4 (8.3)	0.706	12 (12.2)	7 (7.1)	0.166
Problem Areas in Diabetes(PAID) [b]									
Raw score, Mean (SD)	11.2 (11.9)	6.0 (8.3)	0.012	21.1 (11.7)	11.5 (8.6)	<0.001	16.1 (12.7)	8.8 (8.8)	<0.001
Proportion with raw score ≥ 40, n (%)	2 (4.0)	1 (2.0)	0.564	5 (10.4)	0 (0.0)	0.025	7 (7.1)	1 (1.0)	0.034
Percentage score, Mean (SD)	14.0 (14.9)	7.6 (10.3)	0.012	26.4 (14.6)	14.3 (10.7)	<0.001	20.1 (15.9)	11.0 (11.0)	<0.001
WHO (Five) Well-Being Index [c]									
Raw score, Mean (SD)	19.1 (5.2)	20.0 (5.1)	0.514	17.6 (4.7)	16.1 (4.1)	0.049	18.4 (5.0)	18.0 (5.0)	0.382
Proportion with raw score < 13, n (%) Poor well being	6 (12.0)	4 (8.0)	0.739	7 (14.6)	10 (20.8)	0.366	13 (13.3)	14 (14.3)	0.655
Percentage score, Mean (SD)	76.5 (20.8)	80.1 (20.5)	0.514	70.6 (18.6)	64.4 (16.6)	0.049	73.6 (19.9)	72.2 (20.1)	0.382
Proportion with percentage score<25, n (%) Likely depression	1 (2.0)	1 (2.0)	0.317	4 (8.3)	0 (0.0)	0.046	5 (5.1)	1 (1.0)	0.180
Self-Efficacy for Diabetes (DSEQ) [d], Mean (SD)	68.7 (16.9)	79.8 (10.2)	0.001	70.7 (12.9)	69.1 (10.0)	0.449	69.7 (15.0)	74.4 (11.4)	0.027
**Questionnaire Score**	**Malawi Baseline** N = 50	**Malawi Follow-up** N = 47	P-value*	**Mozambique Baseline** N = 48	**Mozambique Follow-up** N = 48	P-value*	**Overall Baseline** N = 98	**Overall Follow-up** N = 95	P-value*
Medical Outcomes Study: (SF-20) [e]									
Functioning									
Physical functioning									
Median (IQR)	91.6 (75-100)	100 (91.6-100)	0.545	100 (100-100)	100 (95.8-100)	0.009	100 (83.3-100)	100 (91.6-100)	0.282
Role functioning									
Median (IQR)	100 (50-100)	100 (100-100)	0.084	100 (100-100)	100 (100-100)	0.183	100 (100-100)	100 (100-100)	0.030
Social functioning									

For peer review only - http://bmjopen.bmj.com/site/about/guidelines.xhtml

Questionnaire Score	Malawi Baseline N = 50	Malawi Follow-up N = 47	P-value*	Mozambique Baseline N = 48	Mozambique Follow-up N = 48	P-value*	Overall Baseline N = 98	Overall Follow-up N = 95	P-value*
Median (IQR)	100 (100-100)	100 (100-100)	0.813	100 (100-100)	100 (100-100)	0.542	100 (100-100)	100 (100-100)	0.793
Well-Being									
Mental health									
Median (IQR)	88 (76-96)	92 (84-100)	0.069	80 (68-88)	76 (64-84)	0.681	84 (72-88)	84 (72-92)	0.344
Health perceptions									
Median (IQR)	46.8 (32.2-60)	65 (41.8-90)	0.000	53.6 (30 -67.2)	48.6 (32.2-61.1)	0.894	46.8 (30-62.2)	56.8 (36.8-76.8)	0.005
Pain									
Median (IQR)	40 (0-60)	20 (0-60)	0.284	40 (0-60)	40 (20-60)	0.407	40 (0-60)	20 (0-60)	0.171

* P-value calculated using McNemar's test or paired ttest or non-parametric Wilcoxon ranksum test

a PHQ-9; responses were for over the last two weeks; scale for all responses ranges from 0 = Not at all to 3=Nearly every day; total scores range from 0 to 27 with higher scores indicating poor health status.

b PAID: Problem Areas in Diabetes; scale for all responses ranges from 0 = not a problem to 4 = serious problem; total scores range from 0 to 80. The score is multiplied by 1.25 to get the percentage score and a score of >=40 is severe diabetes distress

c WHO Well-Being Index; responses were for over the last two weeks; scale for all responses ranges from 0=At no time to 5=All of the time; total raw scores range from 0 to 25, 0 representing worst possible and 25 representing best possible quality of life. The raw score is multiplied by 4 to obtain a percentage score ranging from 0 to 100.

d Self-Efficacy for Diabetes (Modified version of DSEQ); scale for all responses ranges from 1 = Not at all confident to 10 = Totally confident; total scores range from 9 to 90 with higher scores indicating higher self-efficacy.

e SF-20; Physical functioning total raw score=18, Role functioning total raw score=6, Social functioning total raw score=6, Mental health total raw score=30, Health perception total raw score=25, Pain total raw score=6. All parameters scaled 0-100. A higher score indicates better functioning or well-being. The only exception is pain: a higher score indicated more pain.

nb:Mean (SD) expressed for normally distributed variables and median (IQR) for non-normally distributed variables.

For peer review only

For peer review only - http://bmjopen.bmj.com/site/about/guidelines.xhtml

Qualitative findings (Table 4)

Sixty-six individuals were interviewed. Overall,participants who took part shared that the EXTEND programme had a positive impact on their behaviour, indicating improvements in lifestyle habits, including increasing physical activity, and improving food choices. They also reported improvements regarding losing weight and taking medication as advised by their doctor (Table 4). Participants on a whole expressed positive experience attending a DSME programme such as EXTEND and emphasised a strong need for such self-management education in their local communities. Due to lack of information and education about the management of T2D, the people with this condition had often been misinformed. They had received inconsistent messages about why they have diabetes, what food they should eat, what types of alcohol they should drink or avoid and so on. In addition to the inconsistent information provided to them, they had also been exposed to confusing and conflicting advice from clinicians highlighting that education in diabetes should not only be available for patients, but for healthcare professionals also a detailed analysis of this qualitative study is reported elsewhere.

For peer review only - http://bmjopen.bmj.com/site/about/guidelines.xhtml

Table 4: Qualitative data from patients who received the DSME

Behaviour Change	Patient Perspective
Improvements in taking medication	*I am a living example when I was first diagnosed with T2D I was prescribed to take metformin in the morning and evening but after learning about my condition especially diet topic I was able to manage my diet and as a result I was asked to take metformin once a day and as of now I am only taking half a tablet in the morning and another half in the evening (P5, non-participant, Malawi)*
Increasing physical activity	*For me, this training program was a privilege, and I've been capitalizing on everything I've learned. Especially in the alignment between eating and physical exercise, because this is where I had many problems (P4, patient, Moz)*
	I could walk from home going to Msungwi but when I reach there I could feel so much pain as if something bad is happening in my body. But after getting the education I have been walking long distances during the evening and I have seen that its working (P5, female patient, Malawi)
	The other part which I also liked most was the advice we get from the clinic such as doing different physical exercises like moving a wheel bar, cultivating the garden, so this is helping our bodies to be strong (P6, male patient, Malawi)
Improving food choices	*We use to abuse on the oil, tomato, onion, everything. Now I know how to do things moderately. I learned a lot, the course was valuable (P4, patient, Moz)*
	For me I think the program was good because they taught us how to take care of our bodies and the need to consume food that has less cholesterol (P2, female patient Malawi)
	We were just ignorant especially on the issue of diet and this was not helping us, but now after getting the education we are able to take care of ourselves (P2, female patient Malawi)
	Mmm, and the foods we were taught to eat it's our locally Malawian foods; they didn't tell us to take foreign food which we could spend thousands of money to buy, no so the examples were relevant (P2, female patient Malawi)
Manage stress	*We stopped having stress and we accepted that we are T2D patients. You also taught us how we can live long by doing exercise, having good diet and etc. The main thing is to accept and avoid stress (P5, male patient, Malawi)*
Losing Weight	*I was weighing over 105kgs but now I have reduced the weight to 92 (P6, female patient Malawi)*
	I was weighing 85kgs but am weighing 75 kgs and I feel very light now (P1, male patient Malawi)
	I already lost 4 kg. My blood sugar levels have already dropped. The tiredness I used to feel I no longer feel (P1, patient, Moz)

Data sharing statement

There are no other data available

<u>Discussion</u>

This study demonstrates that it is possible and acceptable to deliver the culturally, contextually and linguistically adapted EXTEND programme in an urban setting in two LMIC. A study to recruit to and collect data for the purpose of evaluating the biomedical and psychological impact of the EXTEND programme was successfully developed and implemented.

A number of key learnings came from this feasibility study. Firstly, additional health information should be collected from participants including; medicine adherence, access to medicine, engagement with traditional healers and use of traditional medicines, previous education in diabetes

For peer review only - http://bmjopen.bmj.com/site/about/guidelines.xhtml

and the presence of any communicable disease comorbidities i.e. HIV/AIDS. The incomplete objective physical activity data at both sites means that future collection will require dedicated 'hands-on' technology support for device set-up, initialisation and download. This is particularly important as increased objectively measured physical activity in LMICs will progress the field of physical activity surveillance and intervention development[20].

It was anticipated that the development of the protocol would take approximately 12 weeks however it took 26 weeks therefore a longer time-frame for the development of these core documents should be built into any future work. Due to the large interest and requests from people with T2D for the DSME programme (after recruitment had ceased) as reported by local research teams it is preferable that a future effectiveness study focuses on less traditional study designs, for example step-wedged or wait lists, or build in infrastructure/finance to permit control arm participants to receive the programme at the end of the study at no personal cost to them.

The baseline data indicated people with T2D in the two urban settings have poorer glycaemic control than their UK counterparts[21]. This difference extends to the pooled baseline HbA1c from the USA, Sweden and Thailand reported in a systematic review conducted by Steinsbekk and colleagues in 2012, [22]. It is well recognised that health outcomes for patients with T2D are largely dependent on the individual's ability to effectively implement and sustain complex self-management skills into their daily lives [22 23]. Encouragingly, whilst this feasibility study was not powered to detect a change in outcomes at 6 months both clinically and statistically significant changes in biomedical and psychological outcomes are observed. The reduction in HbA1c, for example, that is approaching 1% is clinically meaningful for example a 1% reduction in HbA1c reportedly reduces the risk for any end point related to diabetes by 21% [24]. Further, reductions in SBP and Diastolic Blood Pressure (DBP) of ≥2 mm Hg are reported to significantly reduce the incidence of CVD [25 26], thus the reduction of -5.9 and -6.1 (for SBP and DPB, respectively) can be considered clinically meaningful. These data are in-line with evidence from previously tested DSME programmes in HIC [22]. Our data also indicate that these changes are not

For peer review only - http://bmjopen.bmj.com/site/about/guidelines.xhtml

driven by weight loss as one might expect but may be instead attributed to the reported lifestyle changes (Table 4) and a greater understanding of their condition and thus adherence to both glucose lowering and anti-hypertensive medications.

The improvements observed in self-efficacy and knowledge of diabetes are supported by previously reported DSME [27 28] and is the core for successful self-management. Behaviour change is not determined solely by knowledge and information, it is however, fundamental that the individual understands their condition and is equipped with the appropriate skills and confidence to self-manage. The improved diabetes distress score suggests that attending a self-management programme could have a positive impact on behaviour change and emotional wellbeing (PAIDS) [29]. The data from the qualitative study support the acceptability and need for a DSME such as EXTEND in LMICs, given the lack of available education around T2D and their limited access to support for the management of their diabetes.

The rising prevalence of T2D in LMICs, whose healthcare systems are already under immense pressure with infectious disease and limited resources, highlights the need for low-cost effective interventions. This feasibility study demonstrated short-term benefits of the DSME EXTEND that meets international principles for self-management education. To our knowledge this is the first DSME meeting international guidelines for DSME in Malawi and Mozambique. A definitive trial that includes multiple settings (urban, rural and remote) and cost outcomes is required to formally evaluate the effectiveness and cost-effectiveness of this DSME, and be powered to examine impact on clinical outcomes, diabetes complications with adequate follow-up to explore the persistence of any changes observed in outcome measures. It should also address the sustainability of such programmes in the settings they are tested with implementation pathways and buy-in from influential stakeholders and national decision makers from the off-set. It is advisable to employ the 'train the trainer' model and use lay educators which has been shown to be as effective as HCP provision in the UK [30].

For peer review only - http://bmjopen.bmj.com/site/about/guidelines.xhtml

Study limitations include it was only delivered in urban settings thus results are not generalizable to the wider diabetes populations e.g. rural and remote dwellers. Further, this study was not powered to look at change in biomedical or psychosocial outcomes nor did we have a control group therefore it cannot be ruled out that the observed changes were due to chance or a maturation effect. The qualitative study followed a robust process to collect and analyse data, however, we acknowledge limitations around the sample size and diversity of individuals' characteristics therefore may not be generalizable to other patients with T2D from other parts of either country.Study strengths include use of standardised case report forms (CRF) and standard operating procedures at each site, double entry of all data, the successful recruitment of desired sample and high retention rate. All data were collected at each site for both time-points except objective measure of physical activity. Lastly the significant and clinically relevant reduction in biomedical, psychological parameters and the patient experience demonstrate a need for DSMEs such as EXTEND.

Conclusion

It is feasible to train educators to successfully deliver a fit-for-purpose DSME in urban settings in two LMIC. The positive biomedical and psychosocial outcomes observed warrant the formal evaluation of the effectiveness, cost-effectiveness and sustainability of the EXTEND programme in Malawi and Mozambique.

Funding Statement

This work was funded by the Medical Research Council Global Challenges Research Fund NCDs Foundation Awards 2016 Developmental Pathway (MR/P02548X/1).

Data availability

The authors confirm that the data supporting the findings of this study are available within the article and its supplementary materials. Raw data that support the findings of this study are available from the corresponding author (EMB, MJD) upon reasonable request.

For peer review only - http://bmjopen.bmj.com/site/about/guidelines.xhtml

For peer review only

Author's contribution

EMB: Conceived the study and participated in the study design and co-ordination of the study. Participated in the design of the protocol, case report form and database. Contributed to data cleaning, analysis and interpretation, the development of intervention. EMB drafted the manuscript and co-ordinated and drafting of revisions of manuscript, figures and tables. CB and AM: participated in the study coordination, study design, acquisition of data and interpretation of results, revisions of manuscript. LS: participated in the study coordination, study design, acquisition of data and interpretation of results, revisions of manuscript. JM: Participated in the co-ordination of the study and was involved in the development of intervention, database design, data cleaning and revisions of manuscript. MJD: Conceived the study and participated in the study design, the development of intervention, data analysis and interpretation and contributed to revisions of manuscript. AD: Conceived the study and participated in the study design, designing of the case report form, data interpretation and revisions of manuscript. SS, BS, CT and AR: Contributed to the development and delivery of intervention, data collection and interpretation and to the revisions of manuscript. ACC: Conceived the study and participated in the study design, the development of intervention and Case report form. In addition to the database design, data interpretation and contributed to revisions of manuscript. HN: Participated in writing of the protocol and development of the case report form, development of intervention and revisions of manuscript. DB, JCC, MH, DMH, KK and NL: Conceived the study and participated in the study design, interpretation of results and revision of manuscript GW: Led the statistical analysis, drafting of data tables and contributed to revisions of the manuscript.

Competing Interests

KK reports grants and or personal fees from Amgen, Astrazeneca, Baye, NAPP, Lilly, Merck Sharp and Dohme, Novartis, Novo Nordisk, Roche, Berlin-Chemie AG/Menarini Group, Sanofi-Aventis, Servier, Bohringer Ingelheim and Pfizer outside the submitted work. MJD reports grants from University of Leicester and international development fund during this work. DH reports non-financial support from Novo Nordisk during this work. No other author has any declarations.

Acknowledgments

Individual REDCap study databases and ongoing data management were developed and conducted in partnership with the NIHR Leicester Biomedical Research Centre Bioinformatics Hub.

For peer review only - http://bmjopen.bmj.com/site/about/guidelines.xhtml

References

1. KAISER AB, ZHANG N, DER PLUIJM WV. Global Prevalence of Type 2 Diabetes over the Next Ten Years (2018-2028). *Diabetes* 2018;67(Supplement 1):202-LB. doi: 10.2337/db18-202-LB
2. International Diabetes Federation (IDF) IDF Diabetes Atlas. 8th Edition IDF, Brussels., http://www.diabetesatlas.org/resources/2017-atlas.html. 2017
3. Beagley J, Guariguata L, Weil C, et al. Global estimates of undiagnosed diabetes in adults. *Diabetes research and clinical practice* 2014;103(2):150-60. doi: 10.1016/j.diabres.2013.11.001
4. UK Prospective Diabetes Study (UKPDS). VIII. Study design, progress and performance. *Diabetologia* 1991;34(12):877-90. [published Online First: 1991/12/01]
5. Bommer C, Sagalova V, Heesemann E, et al. Global Economic Burden of Diabetes in Adults: Projections From 2015 to 2030. *Diabetes care* 2018;41(5):963-70. doi: 10.2337/dc17-1962 [published Online First: 2018/02/25]
6. (IDF) IDF. IDF Diabetes Atals 9th Addition. Available at: https://www.diabetesatlas. [Accessed March 2020] 2019
7. Davies MJ, D'Alessio DA, Fradkin J, et al. Management of hyperglycaemia in type 2 diabetes, 2018. A consensus report by the American Diabetes Association (ADA) and the European Association for the Study of Diabetes (EASD). *Diabetologia* 2018;61(12):2461-98. doi: 10.1007/s00125-018-4729-5 [published Online First: 2018/10/06]
8. Federation ID. IDF Clinical Practice Recommendations for Managing Type 2 Diabetes in Primary Care *file:///Z:/Downloads/IDF-T2D-CPR-2017-interactivepdf* 2017
9. NICE. Diabetes in adults (Quaility Standard 6): Available at: https://www.nice.org.uk/guidance/qs6 [Accessed March 2020], 2016.
10. Funnell MM, Brown TL, Childs BP, et al. National standards for diabetes self-management education. *Diabetes care* 2009;32 Suppl 1:S87-94. doi: 10.2337/dc09-S087 [published Online First: 2009/01/06]
11. Stephani V, Opoku D, Beran D. Self-management of diabetes in Sub-Saharan Africa: a systematic review. *BMC public health* 2018;18(1):1148. doi: 10.1186/s12889-018-6050-0 [published Online First: 2018/10/01]
12. Davies MJ, Heller S, Skinner TC, et al. Effectiveness of the diabetes education and self management for ongoing and newly diagnosed (DESMOND) programme for people with newly diagnosed type 2 diabetes: cluster randomised controlled trial. *BMJ* 2008;336(7642):491-95. doi: 10.1136/bmj.39474.922025.BE
13. Gillett M, Dallosso HM, Dixon S, et al. Delivering the diabetes education and self management for ongoing and newly diagnosed (DESMOND) programme for people with newly diagnosed type 2 diabetes: cost effectiveness analysis. *BMJ* 2010;341:c4093. doi: 10.1136/bmj.c4093
14. Khunti K, Gray LJ, Skinner T, et al. Effectiveness of a diabetes education and self management programme (DESMOND) for people with newly diagnosed type 2 diabetes mellitus: three year follow-up of a cluster randomised controlled trial in primary care. *BMJ : British Medical Journal* 2012;344:e2333. doi: 10.1136/bmj.e2333
15. Skinner TC, Carey ME, Cradock S, et al. Diabetes Education and Self-Management for Ongoing and Newly Diagnosed (DESMOND): process modelling of pilot study. *Patient Educ Couns* 2006;64(1-3):369-77. doi: 10.1016/j.pec.2006.04.007 [published Online First: 2006/10/03]
16. Harris PA, Taylor R, Thielke R, et al. Research electronic data capture (REDCap)--a metadata-driven methodology and workflow process for providing translational research informatics support. *J Biomed Inform* 2009;42(2):377-81. doi: 10.1016/j.jbi.2008.08.010 [published Online First: 2008/10/22]
17. Ritchie J SL, O'Connor W. Qualitative research practice: a guide for social science students and researchers. In: Ritchie JaLJe, ed.: London: Sage 2003:76-79.

For peer review only - http://bmjopen.bmj.com/site/about/guidelines.xhtml

18. Gale NK, Heath G, Cameron E, et al. Using the framework method for the analysis of qualitative data in multi-disciplinary health research. *BMC Medical Research Methodology* 2013;13(1):117. doi: 10.1186/1471-2288-13-117
19. Korstjens I, Moser A. Series: Practical guidance to qualitative research. Part 4: Trustworthiness and publishing. *European Journal of General Practice* 2018;24(1):120-24. doi: 10.1080/13814788.2017.1375092
20. Sallis JF, Bull F, Guthold R, et al. Progress in physical activity over the Olympic quadrennium. *Lancet (London, England)* 2016;388(10051):1325-36. doi: 10.1016/s0140-6736(16)30581-5 [published Online First: 2016/08/01]
21. Misra A, Gopalan H, Jayawardena R, et al. Diabetes in developing countries. *J Diabetes* 2019;11(7):522-39. doi: 10.1111/1753-0407.12913 [published Online First: 2019/03/14]
22. Deakin T, McShane CE, Cade JE, et al. Group based training for self-management strategies in people with type 2 diabetes mellitus. *Cochrane Database Syst Rev* 2005(2):CD003417-CD17. doi: 10.1002/14651858.CD003417.pub2
23. Chatterjee S, Davies MJ, Heller S, et al. Diabetes structured self-management education programmes: a narrative review and current innovations. *The lancet Diabetes & endocrinology* 2018;6(2):130-42. doi: 10.1016/S2213-8587(17)30239-5 [published Online First: 2017/09/29]
24. Stratton IM, Adler AI, Neil HA, et al. Association of glycaemia with macrovascular and microvascular complications of type 2 diabetes (UKPDS 35): prospective observational study. *BMJ (Clinical research ed)* 2000;321(7258):405-12. doi: 10.1136/bmj.321.7258.405
25. Wong GWK, Wright JM. Blood pressure lowering efficacy of nonselective beta-blockers for primary hypertension. *Cochrane Database Syst Rev* 2014(2):CD007452-CD52. doi: 10.1002/14651858.CD007452.pub2
26. Wong GWK, Laugerotte A, Wright JM. Blood pressure lowering efficacy of dual alpha and beta blockers for primary hypertension. *Cochrane Database Syst Rev* 2015;2015(8):CD007449-CD49. doi: 10.1002/14651858.CD007449.pub2
27. Deakin TA, Cade JE, Williams R, et al. Structured patient education: the diabetes X-PERT Programme makes a difference. *Diabet Med* 2006;23(9):944-54. doi: 10.1111/j.1464-5491.2006.01906.x
28. Steinsbekk A, Rygg LØ, Lisulo M, et al. Group based diabetes self-management education compared to routine treatment for people with type 2 diabetes mellitus. A systematic review with meta-analysis. *BMC health services research* 2012;12:213-13. doi: 10.1186/1472-6963-12-213
29. Sturt J, Dennick K, Hessler D, et al. Effective interventions for reducing diabetes distress: systematic review and meta-analysis. *International Diabetes Nursing* 2015;12(2):40-55. doi: 10.1179/2057332415Y.0000000004
30. Mandalia PK, Stone MA, Davies MJ, et al. Diabetes self-management education: acceptability of using trained lay educators. *Postgrad Med J* 2014;90(1069):638-42. doi: 10.1136/postgradmedj-2014-132865 [published Online First: 2014/09/27]

For peer review only - http://bmjopen.bmj.com/site/about/guidelines.xhtml

EXTEND Supplementary material 1: CONSORT diagram

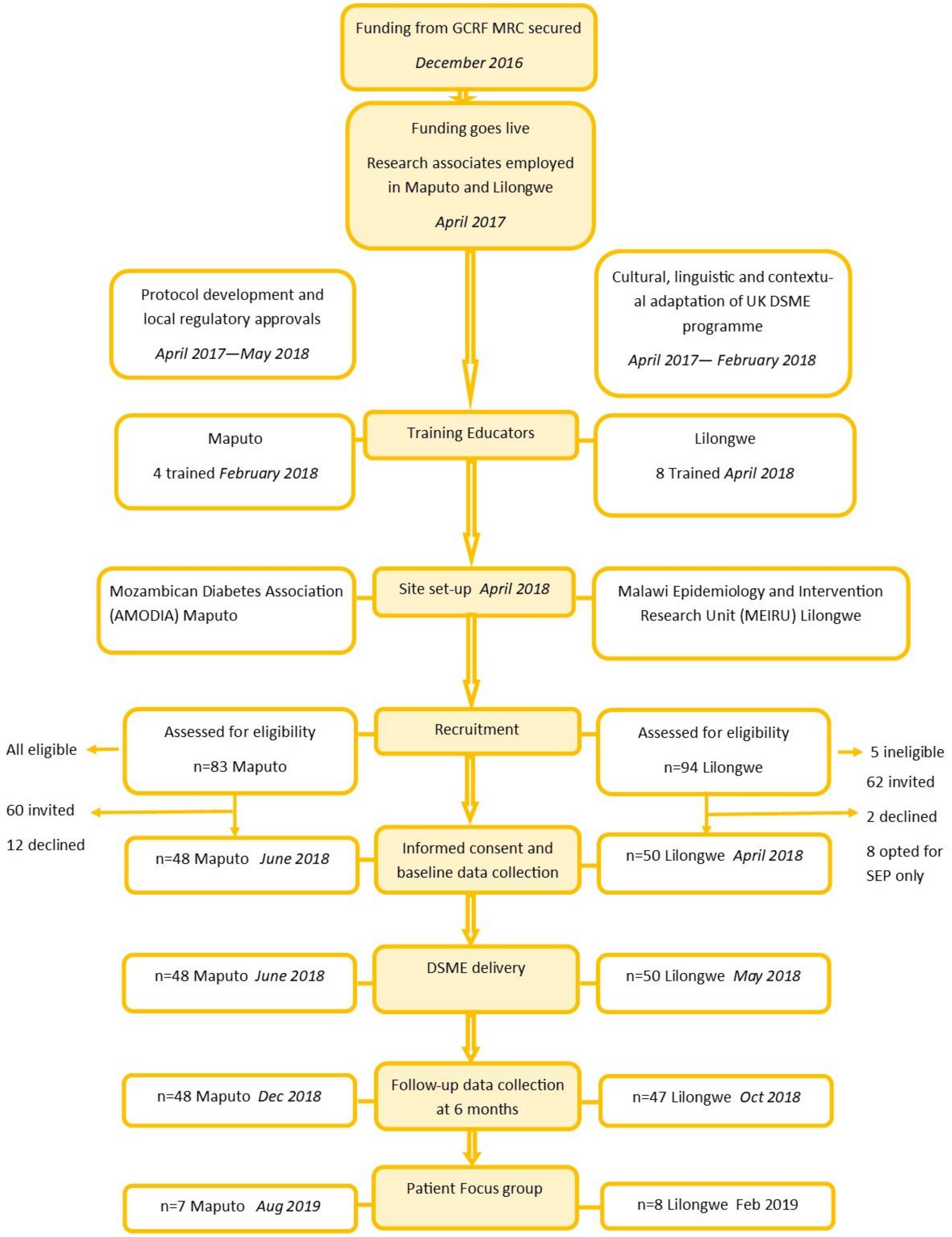

For peer review only - http://bmjopen.bmj.com/site/about/guidelines.xhtml

EXTEND Supplementary material 2: DESMOND programme and EXTEND adaptations

The DESMOND curriculum is broken down into various sessions to help build on the persons understanding of the condition.

The sessions are as follows:
Section A Introduction and Housekeeping

Section B The participant story

Section C Type 2 diabetes and Glucose

Section D Monitoring type 2 diabetes

Section E Food and glucose control

Section F Reflections

Section G Reflections if delivering over 2 days

Section H Long term effects of type 2 diabetes

Section I Physical activity

Section j Food and health

Section K Self-management plan

Section L questions and future care

For peer review only

For peer review only - http://bmjopen.bmj.com/site/about/guidelines.xhtml

Item	Adaptation
Language	Maputo - Portuguese<br>Lilongwe – Chichewa<br>More images used as opposed to words driven by expected low literacy levels (outside the urban areas)<br>The handouts were able to be seen via a mobile devise as printing options were limited to have paper copy of resources
Terminology	Glucose referred to as sugar throughout as this was a more familiar term
Images	Removal and replacement of image irrelevant to local people. This included an image of hovering, and replace with brush for sweeping floors. Add image of washing line and washing clothes by hand because there are no machines available/used.<br><br>Multiple images were provided for blood pressure monitoring and overweight with the group selecting the most appropriate ones that happened to differ from DESMOND.
Diabetes complications Depression	The concept of 'depression' does not exist in these two cultures 'low feelings' / 'low mood' replaced depression. New innovative resources were developed for helping to score the low moods so these were then talked about during the session
Diabetes complications Hypercholesterolemia	LDL should be used & not HDL as the evidence for CV risk is based on LDL levels not HDL.
Diabetes Complications Erectile dysfunction Retinopathy	If a male has erectile dysfunction his partner/wife will believe that this is because he is being unfaithful. This leads to wider martial problems. Therefore a session was added to explain what this health issues is, why it happens and what can be done to improve it.<br><br>Various tools were used when delivering the complications session to include a sieve to explain more fully about retinopathy. The access to routine retinal screening was an issue so again it was about supporting the person living with diabetes to be able to change those things that they had control over or indeed had access to via their local health care system.
Traditional medicine/religious beliefs	A session was added to allow discussion of this topic to explore traditional and folklore and how to understand what would work to manage diabetes and what was not considered relevant to support diabetes. For example in some villages hypoglycaemic events are viewed as witchcraft – in which the person has been possessed and therefore it is a taboo subject.<br><br>Some individuals may seek out the help of a "local Dr /elder in the community "in search of a cure but this isn't as a long term thing as they are expected to pay for this advice and may be given herbs or spices.
Pictorial representation	Signs/symptoms of type 2 diabetes, causes, ways to manage blood sugar, symptoms of highs and lows, how to treat lows(hypos), ways to manage weight, reduce cholesterol, blood pressure,

For peer review only - http://bmjopen.bmj.com/site/about/guidelines.xhtml

<table>
<tr><td></td><td colspan="2">smoking cessation, improve mood, benefits of activity, looking after feet, good and bad fats, a pictorial health profile and action plan</td></tr>
<tr><td>Food sessions</td><td colspan="2">Laminated pictures of food selected and sources by patients and educators examples provided below.</td></tr>
<tr><td></td><td>Malawi food examples
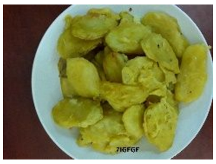
Zigege
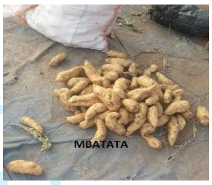

Mbatata
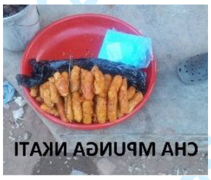
Cha mpunga nkati
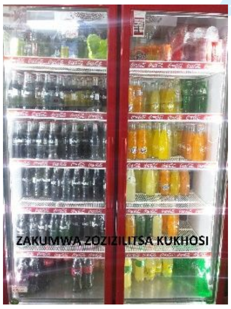

Isohkuk astilizizoz Awmukaz</td><td>Mozambique food examples
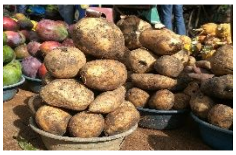
Batata
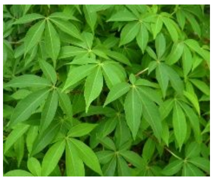
Cassava leaves
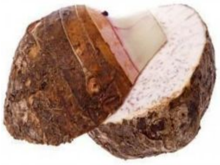
Inhame
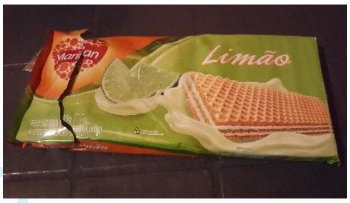

Wafer</td></tr>
<tr><td>Session E Food and glucose control</td><td colspan="2">100 Kcal game chickpeas were used instead of a kit-kat. The discussions were the same except the groups were how long it would take them to walk off each of the items

Carbohydrate and weight management there were no other changes as the basic carbohydrate foods were eaten in both countries so the activity and discussions didn't differ</td></tr>
<tr><td>Session J Food and health</td><td colspan="2">The message regarding fats was simplified to good and bad fat and included pictures of the fats used locally which were laminated. These were included in the fat continuum.</td></tr>
<tr><td>Delivery structure</td><td colspan="2">2*3 hour session was selected by user groups. No whole day sessions were provided.</td></tr>
</table>

For peer review only

For peer review only - http://bmjopen.bmj.com/site/about/guidelines.xhtml

EXTEND Supplementary material 3: Training report Malawi

Running order of the training programme

Core day 1: With Interpreter and Project Coordinator
9.00 - 12.00 Covered: Format of training, Philosophy,Theories, Facilitation Skills and Educator behaviour, Observation tools
12.00 – Onwards: Trainers to prepare room and all resources for Training

Core day 2: With Educators, Interpreter and Project Coordinator
09.00 Introductions/Housekeeping/Outline of week.
09.20 Educators introduce themselves/current role and previous experience of delivering education
09.40 Break
10.00 Your role in this study - delivered by Project Supervisor
10.40 Exploring Philosophy underpinning SEP
11.00 Linking underpinning theories to sessions in the SEP
12.30 Lunch
13.15 Focus on Educator facilitation skills
14.00 Focus on Educator behavior
14.40 Introduction to the curriculum
15.40 Close – Trainers prepare room for tomorrow

Core day 3 with Educators, Interpreter and Project Coordinator
8.30 Welcome back/Housekeeping – what do they remember from yesterday?
09.00 Walk through the Programme – Sessions A-F
10.30 Break
10.40 Walk through the Programme continued– Sessions G- L
12.15 Lunch
13.15 Preparation and Practice – Educators plan their delivery
And introduction of observation tools
15.30 Close and Evaluations

Core Day 4: With Educators, Interpreter and Project Coordinator
08.30 Welcome back/Housekeeping/Purpose of today
08.40 Preparation and practice for delivery
12.00 Lunch
13.00 Preparation of room and further practice
15.30 Close

Delivery to patients – Day 1 with Educators, Interpreter and Project Coordinator
08.00 Educators and Trainers arrive to prepare for session
08.30 Patients arrive
09.00 Programme delivery and observation begins
12.30 End of part 1 of programme - Lunch
13.30 Educator feedback and preparation and practice for next day
15.30 Close

Delivery to patients – Day 2 with Educators, Interpreter and Project Coordinator
08.00 Educators and Trainers arrive to prepare for session
08.30 Patients arrive
09.00 Programme delivery and observation begins
12.30 End of part 2 of programme
13.30 Educator feedback and ongoing support of Educator development
15.30 Close

For peer review only - http://bmjopen.bmj.com/site/about/guidelines.xhtml

Introduction

As part of the EXTEND Study 2 Trainers from the Leicester Diabetes Centre in the United Kingdom (UK) visited Lilongwe in June 2017 to deliver a UK based SEP to patients and to take feedback on how it could be adapted and translated to be more culturally sensitive to the target population in Lilongwe.

In February 2018 the 2 Trainers returned with the adapted curriculum and its associated resources with the intention of training local Educators to be able to deliver to 50 patients during the feasibility study.

It was also an opportunity to identify if the adaptations had been correctly interpreted following the last visit and to explore with Hazel Namadingo about patient engagement to encourage attendance to the programme and ideas on sustainability should the programme go beyond the pilot.

Training Plan

The training had 5 components:

1. Training for the Interpreter and Project Coordinator in the background to the programme and quality assurance.

As the training had to be delivered via an Interpreter it was important that separate training for the Interpreter and the project lead in the underpinning philosophy of the programme was built into the Training Plan. The interpreter and the project lead were also trained on how to use the observation tools as part of the Quality Assurance model for this study.

2. Training for the Educators in the background to the programme

Training the Educators so that they had a clear understanding of how the structured written curriculum and resources should be used. This involved exploring the importance of the underpinning philosophy of the programme and giving them a realistic understanding on the adult learning theories upon which the programme is based and why the sessions have been put together the way they have been.

To complement this - time was also included on facilitation skills and Educator behaviors which would support delivery of the programme using the underpinning philosophy.

3. Training the Educators in how to use the SEP curriculum and associated resources.

All but 2 of the Educators that were to be trained had not observed delivery to patients during the last visit so it was important for the Trainers to ‘walk the Educators through’ each session of the programme, modeling each session to allow for discussion on potential challenges that may occur during delivery and exploring potential strategies to deal with challenges.

4. Supporting Educators to prepare and practice delivery to patients.

Of the 8 Educators who were trained over 3 days, 4 were selected to go forward to deliver in the pilot.

Of these 4 Educators, 2 were patients with diabetes and 2 were Nurses.

These 4 Educators went on to have another days training to allow them to prepare and practice for delivery to patients

For peer review only - http://bmjopen.bmj.com/site/about/guidelines.xhtml

5. Supporting Educators actually delivering to patients via observation and feedback

Although the programme is intended to be delivered by 2 Educators, working together to support each other - for the purposes of the training and because the Educator team had a mix of Health Care Professionals and patients it was decided that they would deliver as a team of 4, at least for the purposes of the training as their practice delivery came the day after completion of training.

The Educators should be able to deliver as a pair for the feasibility study but it's recommended that they should be paired as Health Care Professional with a patient. Should they wish to deliver as a team of 4 this should not affect the study but would obviously impact on Educator capacity.

The delivery of the programme to 10 patients took place over 2 half days and both the Trainers and the Project Coordinator used observational tools to help provide objective feedback to each Educator with regards to key content covered, time management, Educator talk time, the use of Educator specific behaviors and non-verbal behaviors during delivery that linked to the philosophy underpinning the programme

The Educators were also trained and encouraged to use self-reflection tools to support their onward development and were offered access to the Trainers for further support following the visit should that be required, via email or skype.

At the end of the pilot it is hoped themes from the Educators self-reflections can be summarised to identify any learning that could be incorporated into any future Educator training should the SEP be rolled out further.

The Trainers would encourage the Project Coordinator where possible to continue to use the observation sheets which measure key content covered, Educator specific behavior and time management whenever it's feasible for her to observe future Educator delivery – If Possible observing 2 of the 5 SEPs as part of the feasibility study.

Evaluation of the Training - summary feedback received from the Educators

'The training has been good – I liked your facilitation'

'All sessions were great – we learnt new things that will help us teach others and you have taught us very well'

'Our friends with type 2 diabetes will benefit a lot from this SEP and we will work hard to teach others so that type 2 diabetes can be managed

'Thank you for this SEP – we have learned a lot and we will teach others'

'The sessions have been good. Time was managed well – facilitators were very good and friendly'

Evaluation of the Educators first delivery to patients following training using observation tools – summary of results for all 4 Educators

1. Key Content covered – all 4 Educators covered all key content of the sessions they delivered.

For peer review only - http://bmjopen.bmj.com/site/about/guidelines.xhtml

2. Specific Educator behaviors (e.g. Open questions/Reflections and Summaries) – all 4 Educators frequently used open questions, reflections and summaries as well as analogies , examples and visual aids to engage with patients to support systematic learning.

3. Nonverbal Educator behaviors (e.g. eye contact/smiling/open body language) –all 4 Educators displayed positive nonverbal behaviors to support patient engagement throughout all sessions.

4. Educator Talk Time using DESMOND Observation Tool – all 4 Educators achieved the talk time targets based on delivery in English even though they were delivering in Chichewa

5. Time management – the timing of session delivery was generally very well adhered to throughout the whole 6 hour programme.

Summary

The UK Training team adapted the training they normally deliver in the UK to concentrate on specific behaviors and facilitation skills they wanted the Educators to use to support the underpinning philosophy of the SEP when they delivered.They also built in more time for the Trainers to model each of the sessions to allow the curriculum to 'come to life'.

Although only 4 of the 8 Educators trained will be delivering in the pilot – the 4 who will not be will take their knowledge and awareness of the programme back into their local communities and local groups that they engage with . Hopefully these ambassadors will encourage patient recruitment to attend this SEP during the pilot and, if successful, potentially beyond.

By including the Project Coordinator in the training and in particular in the observations of delivery it was felt this would provide extra support in the Educators onward journey when the UK team had left.

In addition the Trainers wanted the Educators to take ownership of their curriculum and so Educators were encouraged to highlight if there were errors in the curriculum or where there were more suitable analogies, food examples etc. that would be more appropriate for their local audience.

What the team in Lilongwe did in planning for 2 practice deliveries to patients by the Educators – one whilst the UK Trainers were there and one a week later - will undoubtedly have supported Educators confidence for when they deliver in the pilot. In addition there were also plans for other ways the Educators could practice delivery in other areas in the time between completion of training and delivery in the pilot.

The Trainers will share these ideas with the other EXTEND site in Maputo, Mozambique.

Acknowledgements

We would like to thank Mia Crampin (Acting Director MEIRU) and Hazel Namadingo (Project Supervisor) for arranging the visit, including logistics and supporting our training plan throughout and beyond.

We would also like to thank Catherine Bamuya (Project Coordinator) for her enthusiasm particularly in the support she helped us with in the observations and to all the individuals who contributed to helping with interpreting throughout our visit especially Veronica.

For peer review only

For peer review only - http://bmjopen.bmj.com/site/about/guidelines.xhtml

Last but not least thanks to all the 8 Educators we trained for their enthusiasm and commitment who are moving forward to delivering in the pilot and to the patients who attended the SEP during our visit.

Appendices

Appendix 1

Part 2: Content Assessment Tool

Educator Name: ______ Date: ______

SESSION B: The Participant Story (40 mins)			
Start:	Finish:	Time taken:	✔ or ✖
Identifies individual participant stories by asking:			
How long do they believe they have had diabetes?			
How did they find out they had diabetes/any symptoms?			
What do they believe caused their diabetes?			
What do they believe are the long-term effects of having diabetes?			
What do they believe are the treatments for their diabetes?			
What is one key question, you would like answering before the end of the course?			
		Score	/6
Assessment Comments			

Appendix 2

Part 3: DESMOND Observational Tool (DOT)

Assessing Educator Talk Time Tool - **Must complete Session C or H plus one other from Sessions C, E, H, J or K**

Educator Name: Date:

DOT assessment			
Session:	Educator Talking:	Participant talking:	Miscellaneous:
Totals:	Total A:	Total B:	Total C:
(Total A)	÷ (Total A+B+C)	= x 100 = Score:	%
Session:	Educator Talking:	Participant talking:	Miscellaneous:
Totals:	Total A:	Total B:	Total C:
(Total A)	÷ (Total A+B+C)	= x 100 = Score:	%
Session:	Educator Talking:	Participant talking:	Miscellaneous:
Totals:	Total A:	Total B:	Total C:
(Total A)	÷ (Total A+B+C)	= x 100 = Score:	%

Session Target	Educator Speaking Below:	Session Target	Educator Speaking Below:
C: Type 2 Diabetes and Glucose	65%	J: Focus on Fat	55%
E: Food Choices: Glycaemia and Insulin Resistance	55%	K: Diabetes Self-Management Plan	50%
H: Long-Term Effects of Type 2 Diabetes	65%		

For peer review only - http://bmjopen.bmj.com/site/about/guidelines.xhtml

Appendix 3

Educator Self Reflection sheet

**What went well?**
**What didn't go so well?**
**What will you do about it?**

For peer review only

For peer review only - http://bmjopen.bmj.com/site/about/guidelines.xhtml

Appendix4

Training Evaluation Sheet

What session did you find most useful & why?
What would you have liked us to spend more time on?
Any other comments

For peer review only

For peer review only - http://bmjopen.bmj.com/site/about/guidelines.xhtml

Supplementary material 4: Data collection procedures for demographic, clinical, bio-anthropometric characteristics and psychological wellbeing.

Medical History

Participants were advised to bring their health passports to both measurement sessions to assist with medical history. The following data were taken from the health passport and/or self-reported by the participant

Medical History	
Co-morbidities	Non-communicable Diseases Questionnaire (NCDQ) [1]
Diabetes Duration	Year of TD2 diagnosis for duration to be calculated (Q6042- Q6045 WHO Survey)[2]
Current medication	Taken from participant's health passports or self -report. Anti-viral medication use was to be collected on a separate CRF and stored separately to both the contacts form and main CRF (link anonymised using the unique Participant Identification number (PID)
Smoking status	Do you currently smoke any tobacco products such as cigarettes, cigars, or pipes?" and answer "Daily, yes not daily, not at all" (Q4000 WHO Survey)[2]
Alcohol	Alcohol Consumption captured using the eight questions in the STEPs survey [3]
Family history of diabetes	Provided list of family members (i.e. mother, father, siblings, paternal and maternal grandmother and grandfather) and asked whether any of them had diagnoses of T1D or T2D over their lifetime

Patient outcomes

Demographics	
Age	Years
Sex	Male or female
Ethnicity	*What is your [ethnic group / racial group / cultural subgroup / others] background*? Answers will come from a list of *relevant response options. (*Q1011 WHO Survey [2])
	Collect first language *"What is your mother tongue?" (*Q1000 of WHO Survey [2])
Socio-economic status	"What is the highest level of education you have completed?" (list of categories from Q1009 WHO Survey [2])
	Occupation (Q1012- Q1014 WHO Survey [2])
	Urban/rural home location will be ascertained based on address of participant

Bio- Anthropometric measurements	
Height	Measured to the nearest 0.1 cm using a portable stadiometer
Weight	Measured to the nearest 0.1 kg using a clinically approved weighing scale
Body mass index (BMI)	Calculated as weight (kg)/height (m^2)
Waist circumference	Measured with an inelastic anthropometry tape to the nearest 0.1 cm at the midpoint between the lower costal margin and iliac crest
Hip circumference	Measured at the level of the greatest protrusion of the gluteal (buttock) muscles whilst ensuring that the tape was not too tight or too loose, was lying flat on the skin, and horizontal. The participant stood erect with their weight evenly distributed on both feet and legs slightly parted, making sure not tense the gluteal (buttock) muscles

For peer review only - http://bmjopen.bmj.com/site/about/guidelines.xhtml

fasting venous blood sample	Taken by a trained Health Care Professional (HCP). Full-blood count, HbA1c, triglycerides and LDL cholesterol was measured from this sample
Blood pressure	Measured using an automated sphygmomanometer with an appropriate sized cuff while the patient was seated, and having rested quietly for 5 minutes. Three measurements were obtained for blood pressure with the average of the last two used in analysis

Health and wellbeing	
Depression	The Patient Health Questionnaire (PHQ-9) [4,5]
Diabetes related distress	PAID (Problem Areas in Diabetes Questionnaire (PAID) short form [6]
Quality of Life	The MOS short-form quality of life survey [7]
Mood	WHO-5 questionnaire commonly used to measure mental wellbeing [8]
Self-efficacy	The Self-efficacy for diabetes questionnaire is a reliable and valid 8-item scale tested in adult with diabetes [9]

Lifestyle behaviours	
Physical activity	Participants answered basic questions on physical activity from the WHO Survey (Q4030- Q4038). To supplement this, participants will also be asked to wear a GENEactiv accelerometer on their non-dominant wrist continuously (i.e. 24 hours a day) for 7 days. The devices were initialised before and downloaded after each use. Participants returned the device to the clinic at the DSME session at baseline. At follow-up they were given a stamped addressed envelope to mail it back to the research team.
Diet composition	Dietary habits were queried using questions from the Malawi STEPS survey (2009). Participants will be asked to report the number of days in the last week they have consumed at least one piece of fruit, at least one piece of veg, the number of fruit servings per day on an average day and the number of veg servings per day on an average day.

References

1. Polonsky WH, Anderson BJ, Lohrer PA, et al. Assessment of diabetes-related distress. *Diabetes care* 1995; **18**(6): 754-60.
2. https://www.who.int/healthinfo/survey/en/. 2004.
3. http://www.who.int/chp/steps/malawi/en/. 2009.
4. Kroenke K, Spitzer RL, Williams JB. The PHQ-9: validity of a brief depression severity measure. *Journal of general internal medicine* 2001; **16**(9): 606-13.
5. van Steenbergen-Weijenburg KM, de Vroege L, Ploeger RR, et al. Validation of the PHQ-9 as a screening instrument for depression in diabetes patients in specialized outpatient clinics. *BMC health services research* 2010; **10**: 235.
6. McGuire BE, Morrison TG, Hermanns N, et al. Short-form measures of diabetes-related emotional distress: the Problem Areas in Diabetes Scale (PAID)-5 and PAID-1. *Diabetologia* 2010; **53**(1): 66-9.
7. Stewart AL, Hays RD, Ware JE, Jr. The MOS short-form general health survey. Reliability and validity in a patient population. *Medical care* 1988; **26**(7): 724-35.
8. https://www.psykiatri-regionh.dk/who-5/Pages/default.aspx.
9. J S. Validity and reliability of the DMSES UK: a measure of self-efficacy for type 2 diabetes self-management. *Primary Health Care Research & Development* 2009: 1-8.

For peer review only - http://bmjopen.bmj.com/site/about/guidelines.xhtml

EXTEND Supplementary material 5: **Other medical history and family medical history**

Characteristics	Malawi n = 50	Mozambique n = 48	Overall n = 98
**Medical history**			
Wheezing/whistling chest, n (%)	2 (4·0)	4 (8·3)	6 (6·1)
Wheezing short of breath	4 (8·0)	2 (4·2)	6 (6·1)
Asthma/bronchitis, n (%)	4 (8·0)	6 (12·5)	10 (10·2)
**Family Medical History**			
Cardiovascular disease, n (%)	0 (0·0)	7 (14·6)	7 (7·1)
Stroke, n (%)	4 (8·0)	12 (25·0)	16 (16·3)
High blood pressure, n (%)	20 (40·0)	33 (68·8)	53 (54·1)
High cholesterol, n (%)	1 (2·0)	3 (6·3)	4 (4·1)
Gestational diabetes, n (%)	1 (2·0)	0 (0·0)	1 (1·0)
Type 1 Diabetes, n (%)	7 (14·0)	0 (0·0)	7 (7·1)
Type 2 Diabetes, n (%)	17 (34·0)	28 (58·3)	45 (45·9)
Depression, n %)	0 (0·0)	4 (8·3)	4 (4·1)
Sleep Disorder, n (%)	0 (0·0)	6 (12·5)	6 (6·1)

Please note: A diagnosis of depression was not collected from participants· % percent

For peer review only - http://bmjopen.bmj.com/site/about/guidelines.xhtml

EXTEND Supplementary material 6: Type of medication

	Baseline			Follow-up		
**Type of Medication**	**Malawi**	**Mozambique**	**Overall**	**Malawi**	**Mozambique**	**Overall**
	n = 50	n = 48	n = 98	n = 47	n = 47	n = 94
Diet and life style only	None	None	None	None	None	None
Mono or combination therapy, n (%)						
Mono therapy						
Metformin	4 (8·0)	25 (52·1)	29 (29·6)	6 (12·8)	12 (25·5)	18 (19·1)
Sulfonylurea	1 (2·0)	5 (10·4)	6 (6·1)	0 (0·0)	3 (6·4)	3 (3·2)
Insulin	0 (0·0)	5 (10·4)	5 (5·1)	0 (0·0)	6 (12·8)	6 (6·4)
Dual therapy						
Metformin + Sulfonylurea	45 (90·0)	5 (10·4)	50 (51·0)	41 (87·2)	12 (25·5)	53 (56·4)
Metformin + Insulin	0 (0·0)	8 (16·7)	8 (8·2)	0 (0·0)	14 (29·8)	14 (14·9)
Anti-hypertension medication, n (%)	36 (72·0)	29 (60·4)	65 (66·3)	31 (62·0)	32 (66·7)	63 (67·0)
Diuretics	26 (52·0)	22 (45·8)	48 (49·0)	16 (34·0)	19 (40·4)	35 (37·2)
Calcium channel blocker	22 (44·0)	19 (39·6)	41 (41·8)	25 (53·2)	20 (42·6)	45 (47·9)
ACE-Inhibitor	8 (16·0)	16 (33·3)	24 (24·5)	3 (6·4)	15 (31·9)	18 (19·1)
Beta blocker	4 (8·0)	8 (16·7)	12 (12·2)	3 (6·4)	7 (14·9)	10 (10·6)
Centrally acting anti-hypertensive	0 (0·0)	1 (3·5)	1 (1·0)	1 (2·1)	2 (4·3)	3 (3·2)
Aspirin	1 (2·0)	0 (0·0)	1 (1·0)	1 (2·1)	0 (0·0)	1 (1·1)
Lipid lowering medication, n (%)						
Statins (Simvastatin)	0 (0·0)	13 (27·1)	13 (13·3)	0 (0·0)	12 (25·0)	12 (12·8)

Please note: Medication adherence was not collected· Data provided count (%)

For peer review only

For peer review only - http://bmjopen.bmj.com/site/about/guidelines.xhtml

CONSORT 2010 checklist of information to include when reporting a pilot or feasibility trial*

Section/Topic	Item No	Checklist item	Reported on page No
**Title and abstract**			
	1a	Identification as a pilot or feasibility randomised trial in the title	Page 1
	1b	Structured summary of pilot trial design, methods, results, and conclusions (for specific guidance see CONSORT abstract extension for pilot trials)	Page 1
**Introduction**			
Background and objectives	2a	Scientific background and explanation of rationale for future definitive trial, and reasons for randomised pilot trial	Pages 4 and 5
	2b	Specific objectives or research questions for pilot trial	Page 6
**Methods**			
Trial design	3a	Description of pilot trial design (such as parallel, factorial) including allocation ratio	Page 6
	3b	Important changes to methods after pilot trial commencement (such as eligibility criteria), with reasons	N/A
Participants	4a	Eligibility criteria for participants	Page 7
	4b	Settings and locations where the data were collected	Page 7
	4c	How participants were identified and consented	Page 7
Interventions	5	The interventions for each group with sufficient details to allow replication, including how and when they were actually administered	Pages 6 and 7
Outcomes	6a	Completely defined prespecified assessments or measurements to address each pilot trial objective specified in 2b, including how and when they were assessed	Page 9 and supplemental material
	6b	Any changes to pilot trial assessments or measurements after the pilot trial commenced, with reasons	N/A
	6c	If applicable, prespecified criteria used to judge whether, or how, to proceed with future definitive trial	N/A
Sample size	7a	Rationale for numbers in the pilot trial	Page 7
	7b	When applicable, explanation of any interim analyses and stopping guidelines	N/A
Randomisation:			
Sequence generation	8a	Method used to generate the random allocation sequence	N/A
	8b	Type of randomisation(s); details of any restriction (such as blocking and block size)	N/A
Allocation concealment	9	Mechanism used to implement the random allocation sequence (such as sequentially numbered containers), describing any steps taken to conceal the sequence until interventions were assigned	N/A

For peer review only

mechanism			
Implementation	10	Who generated the random allocation sequence, who enrolled participants, and who assigned participants to interventions	N/A
Blinding	11a	If done, who was blinded after assignment to interventions (for example, participants, care providers, those assessing outcomes) and how	N/A
	11b	If relevant, description of the similarity of interventions	N/A
Statistical methods	12	Methods used to address each pilot trial objective whether qualitative or quantitative	Page 11 and supplemental material
**Results**			
Participant flow (a diagram is strongly recommended)	13a	For each group, the numbers of participants who were approached and/or assessed for eligibility, randomly assigned, received intended treatment, and were assessed for each objective	Supplemental material
	13b	For each group, losses and exclusions after randomisation, together with reasons	Page 12 and supplemental material
Recruitment	14a	Dates defining the periods of recruitment and follow-up	Page 7 and supplemental material
	14b	Why the pilot trial ended or was stopped	N/A
Baseline data	15	A table showing baseline demographic and clinical characteristics for each group	Page 13
Numbers analysed	16	For each objective, number of participants (denominator) included in each analysis. If relevant, these numbers should be by randomised group	Page 13, 16-18
Outcomes and estimation	17	For each objective, results including expressions of uncertainty (such as 95% confidence interval) for any estimates. If relevant, these results should be by randomised group	Page 13, 16-18
Ancillary analyses	18	Results of any other analyses performed that could be used to inform the future definitive trial	Supplemental material
Harms	19	All important harms or unintended effects in each group (for specific guidance see CONSORT for harms)	NA
	19a	If relevant, other important unintended consequences	NA
**Discussion**			
Limitations	20	Pilot trial limitations, addressing sources of potential bias and remaining uncertainty about feasibility	Page 3, 22-23
Generalisability	21	Generalisability (applicability) of pilot trial methods and findings to future definitive trial and other studies	Pages 20-22
Interpretation	22	Interpretation consistent with pilot trial objectives and findings, balancing potential benefits and harms, and considering other relevant evidence	Pages 22-23

For peer review only

	22a	Implications for progression from pilot to future definitive trial, including any proposed amendments	Pages 22-23
**Other information**			
Registration	23	Registration number for pilot trial and name of trial registry	NA
Protocol	24	Where the pilot trial protocol can be accessed, if available	NA
Funding	25	Sources of funding and other support (such as supply of drugs), role of funders	Page 23
	26	Ethical approval or approval by research review committee, confirmed with reference number	Page 6

Citation: Eldridge SM, Chan CL, Campbell MJ, Bond CM, Hopewell S, Thabane L, et al. CONSORT 2010 statement: extension to randomised pilot and feasibility trials. BMJ. 2016;355.
*We strongly recommend reading this statement in conjunction with the CONSORT 2010, extension to randomised pilot and feasibility trials, Explanation and Elaboration for important clarifications on all the items. If relevant, we also recommend reading CONSORT extensions for cluster randomised trials, non-inferiority and equivalence trials, non-pharmacological treatments, herbal interventions, and pragmatic trials. Additional extensions are forthcoming: for those and for up to date references relevant to this checklist, see www.consort-statement.org.

For peer review only
